# Bmps and Id2a Act Upstream of Twist1 To Restrict Ectomesenchyme Potential of the Cranial Neural Crest

**DOI:** 10.1371/journal.pgen.1002710

**Published:** 2012-05-10

**Authors:** Ankita Das, J. Gage Crump

**Affiliations:** Broad CIRM Center, University of Southern California Keck School of Medicine, Los Angeles, California, United States of America; University of Washington, United States of America

## Abstract

Cranial neural crest cells (CNCCs) have the remarkable capacity to generate both the non-ectomesenchyme derivatives of the peripheral nervous system and the ectomesenchyme precursors of the vertebrate head skeleton, yet how these divergent lineages are specified is not well understood. Whereas studies in mouse have indicated that the Twist1 transcription factor is important for ectomesenchyme development, its role and regulation during CNCC lineage decisions have remained unclear. Here we show that two Twist1 genes play an essential role in promoting ectomesenchyme at the expense of non-ectomesenchyme gene expression in zebrafish. Twist1 does so by promoting Fgf signaling, as well as potentially directly activating *fli1a* expression through a conserved ectomesenchyme-specific enhancer. We also show that Id2a restricts Twist1 activity to the ectomesenchyme lineage, with Bmp activity preferentially inducing *id2a* expression in non-ectomesenchyme precursors. We therefore propose that the ventral migration of CNCCs away from a source of Bmps in the dorsal ectoderm promotes ectomesenchyme development by relieving Id2a-dependent repression of Twist1 function. Together our model shows how the integration of Bmp inhibition at its origin and Fgf activation along its migratory route would confer temporal and spatial specificity to the generation of ectomesenchyme from the neural crest.

## Author Summary

A fascinating question of vertebrate development is how a single cell population—the cranial neural crest—creates such different types of structures as the peripheral nervous system and head skeleton. To date, the molecular signals that instruct neural crest cells to develop into head skeleton at the expense of nervous system have remained elusive. One reason why such signals have been difficult to identify is that they may be required at multiple stages of development—such as in the emergence of neural crest cells themselves. In order to overcome this challenge, we developed a transgenic system in zebrafish that allows us to alter signaling precisely at the stage when neural crest cell fates are determined. In so doing, we have found that the early movement of neural crest cells allows them to escape the influence of suppressive signals at their birthplace, which, in turn, sets in motion a cascade that turns off nervous system genes and turns on head skeleton genes. Together, our studies show how the timing of neural crest cell movement plays a major role in biasing early neural crest cells to form the head skeleton.

## Introduction

The neural crest is a transient, migratory cell population that arises at the boundary between the neural and non-neural ectoderm [1]. Although both cranial and trunk neural crest cells differentiate into non-ectomesenchyme derivatives, such as neurons, glia and pigment cells, CNCCs also generate ectomesenchyme derivatives, in particular many of the cartilage-, bone-, and teeth-forming cells of the head [2]. Whereas much is known about how individual non-ectomesenchyme lineages are specified, how the ectomesenchyme lineage is specified remains actively debated [3]. When the ectomesenchyme versus non-ectomesenchyme lineage decision is made during CNCC development also remains unknown. Whereas cultured avian CNCCs can clonally generate both lineages [2], lineage tracing experiments in zebrafish embryos have failed to identify a common precursor [4], [5].

In zebrafish, CNCCs are first apparent within the anterior neural plate border at 10.5 hours-post-fertilization (hpf), when they begin to express *sox10*, *foxd3*, *sox9b*, and *tfap2a*. Within the next few hours, three streams of CNCCs can be seen migrating away from the neural tube to more ventral positions. Starting around 15.5 hpf, ectomesenchyme precursors begin to downregulate early CNCC genes such as *sox10*, *foxd3*, *sox9b*, and *tfap2a* [6], [7] and up-regulate ectomesenchyme-specific genes such as *dlx2a* [8] and *fli1a* [9]. These ectomesenchyme cells then go on to populate a series of pharyngeal arches from which develops the support skeleton of the jaw and gills in zebrafish, and the jaw, middle ear, and larynx in mammals [10]. Whereas *dlx2a* and *fli1a* are uniquely expressed in the ectomesenchyme lineage, Dlx2a appears to be dispensable for ectomesenchyme formation [8] and the function of Fli1a in ectomesenchyme development remains unknown.

One factor critical for ectomesenchyme specification in mouse is the basic-helix-loop-helix (bHLH) transcription factor Twist1. Both the conventional Twist1 knockout and a conditional Twist1 neural-crest-specific (Wnt1-CRE) knockout display defective ectomesenchyme development, including abnormal perdurance of *Sox10* and loss of expression of many arch ectomesenchyme genes [11], [12]. Furthermore, the neural-crest-specific knockout showed severe reductions of the CNCC-derived craniofacial skeleton, although the lower jaw was less affected. Zebrafish have two Twist1 orthologs, with *twist1b* being expressed in early CNCCs and *twist1a* restricted to ectomesenchyme precursors from 16 hpf onwards [13]. Here, we show that these two Twist1 genes function redundantly for zebrafish ectomesenchyme development, with Twist1 depletion resulting in both perdurance of *sox10* and loss of *fli1a* expression.

As Twist1 genes are expressed throughout the early CNCC domain, an important yet unanswered question is how Twist1 function is specifically regulated in ectomesenchyme precursors. Twist1 function can be regulated by post-translational modification (e.g. phosphorylation), as well as choice of dimerization partners. In particular, Inhibitor of differentiation (Id) proteins, which share HLH but not basic DNA-binding domains with bHLH factors, influence Twist1 homodimer versus heterodimer formation by sequestering Twist1 binding partners such as E2A [14], [15]. Id genes are widely expressed in the early neural crest, and Id2 has been shown to promote neural crest at the expense of epidermis in avians [16]. In zebrafish, Id2a has been shown to regulate neuron and glia formation in the retina, albeit non-cell-autonomously, yet its role in CNCC development has not been explored [17]. In this study we find a novel role of Id2a in CNCC lineage decisions, with down-regulation of *id2a* in migrating CNCCs being essential for ectomesenchyme specification.

Upstream signals that specify ectomesenchyme could originate from the ectoderm where CNCCs are born, from the mesoderm along which CNCCs migrate, or from the endoderm/ectoderm upon which CNCCs condense within the pharyngeal arches. Previous studies have suggested roles for Fgf signaling, in particular Fgf20b and Fgfr1, in ectomesenchyme specification in avians and zebrafish [18], [19]. It was further proposed that CNCCs might acquire ectomesenchyme identity upon arrival in the pharyngeal arches, potentially as a result of endoderm-secreted Fgfs [18]. In contrast, lineage-tracing experiments have revealed that CNCCs in zebrafish are largely restricted to single lineages before migration [4], [5], suggesting that ectomesenchyme fates may be specified at their birthplace within the neural plate border ectoderm.

Bmps such as *bmp2b* in zebrafish are prominently expressed in the non-neural ectoderm, where they influence neural crest induction, migration, survival, and differentiation [20], [21], [22]. However, a role for Bmp signaling in ectomesenchyme lineage decisions has not been previously described, likely because of the earlier essential roles of Bmps in neural crest induction [21]. By manipulating Bmp signaling specifically in migratory CNCCs, we have uncovered an additional function of Bmp signaling in restricting ectomesenchyme potential, in part by maintaining high levels of Id2a. We therefore propose a model in which the early migration of CNCCs away from an ectodermal Bmp source results in down-regulation of Bmp activity and *id2a* expression, which in turn promotes Twist1-dependent establishment of ectomesenchyme fates.

## Results

### Twist1a and Twist1b are redundantly required for ectomesenchyme specification in zebrafish

**Figure 1:**
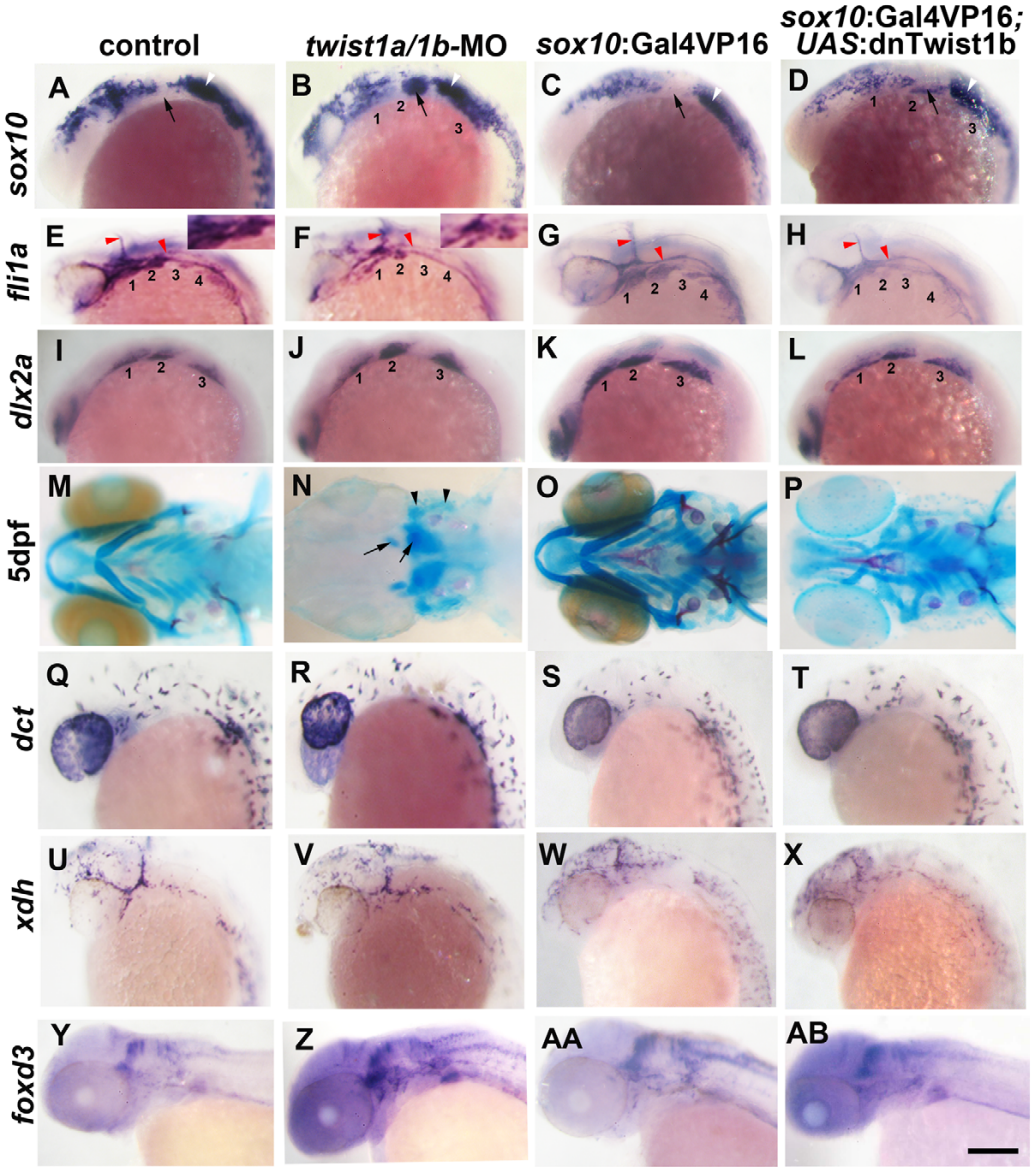
Twist1 genes are required for ectomesenchyme specification in zebrafish. (A–D) Whole mount in situ hybridizations of *sox10* expression at 18 hpf show ectopic expression in the arches (numbered and second arch indicated by black arrow) of *twist1a/1b*-MO (n = 16/16) and *sox10*:Gal4VP16; *UAS*:dnTwist1b (n = 4/4) embryos compared to un-injected (n = 0/14) and *sox10*:Gal4VP16 only (n = 0/9) controls. White arrowheads indicate otic expression. (E–H) Whole mount in situs at 24 hpf show reduction of *fli1a* expression in the arch ectomesenchyme (numbered) of *twist1a/1b*-MO (n = 12/12) and *sox10*:Gal4VP16; *UAS*:dnTwist1b (n = 5/5) embryos compared to un-injected (n = 0/13) and *sox10*:Gal4VP16 only (n = 0/8) controls. Insets in E and F highlight arch ectomesenchyme which is reduced in *twist1a/1b*-MO embryos. Vascular expression of *fli1a* (red arrowheads) is unaffected. (I–L) Whole mount in situs at 18 hpf show a slight reduction of *dlx2a* in *sox10*:Gal4VP16; *UAS*:dnTwist1b embryos (n = 4/4) but not un-injected (n = 0/6), *twist1a/1b*-MO (n = 0/8), and *sox10*:Gal4VP16 only (n = 0/6) embryos. (M-P) Skeletal staining at 5 dpf shows severe loss of CNCC-derived head skeleton in *twist1a/1b*-MO embryos (n = 21/21) and primarily jaw reductions in *sox10*:Gal4VP16; *UAS*:dnTwist1b embryos (n = 9/9) compared to no defects in un-injected (n = 0/24) and *sox10*:Gal4VP16 only (n = 0/16) controls. Whereas only small remnants remain of the CNCC-derived skeleton (arrows), the mesoderm-derived otic capsule cartilage (arrowheads) and posterior neurocranium are less affected in *twist1a/1b*-MO embryos. (Q–T) In situs for *dct* expression at 28 hpf show normal melanophore precursors in un-injected (n = 14), *twist1a/1b*-MO (n = 12), *sox10*:Gal4VP16 only (n = 8), and *sox10*:Gal4VP16; *UAS*:dnTwist1b (n = 8) embryos. (U–X) In situs for *xdh* expression at 28 hpf show normal xanthophore precursors in un-injected (n = 7), *twist1a/1b*-MO (n = 5), *sox10*:Gal4VP16 only (n = 9), and *sox10*:Gal4VP16; *UAS*:dnTwist1b (n = 8) embryos. (Y-AB) In situs for *foxd3* expression at 48 hpf reveal largely normal patterns of glia in un-injected (n = 9), *twist1a/1b*-MO (n = 8), *sox10*:Gal4VP16 only (n = 9), and *sox10*:Gal4VP16; *UAS*:dnTwist1b (n = 8) embryos. Scale bar = 50 µm.

In order to test whether Twist1 genes are required for ectomesenchyme development in zebrafish, we designed translation-blocking MOs to deplete both zebrafish Twist1 orthologs -Twist1a and Twist1b. Injection of *twist1a*-MO or *twist1b*-MO alone resulted in only very subtle changes in *sox10* and no changes in *fli1a*:GFP transgene expression (Figure S1). In contrast, embryos injected with both MOs (*twist1a/1b*-MO) displayed abnormal persistence of *sox10* and reduced expression of *fli1a* in arch CNCCs at 18 and 24 hpf, respectively (Figure 1A, 1B, 1E, 1F). Analysis of doubly transgenic *sox10*:dsRed; *fli1a*:GFP embryos injected with *twist1a/1b*-MO confirmed that CNCCs still formed arches of largely normal size at 28 hpf in the absence of Twist1 function yet failed to initiate *fli1a*:GFP expression (Figure S1). Furthermore, arch expression of *dlx2a* was relatively unaffected in 18 hpf *twist1a/1b*-MO embryos (Figure 1I, 1J). Consistent with the earlier ectomesenchyme gene expression defects, we also found that depletion of both Twist1 proteins resulted in severe reductions of the ectomesenchyme-derived skeleton of the face and anterior neurocranium at 5 days-post-fertilization (dpf) (Figure 1M, 1N). Single depletion of either Twist1a or Twist1b resulted in milder craniofacial defects, particularly in the ventral mandibular and hyoid cartilages (Figure S1). Importantly, injection of a *twist1b* mRNA not targeted by the MOs, but not a control *kikGR* mRNA, partially rescued skeletal defects in *twist1a/1b*-MO embryos, thus showing specificity of MO-generated defects for Twist1 (Figure S1). Hence, despite differences in timing of their expression, Twist1a and Twist1b function redundantly for ectomesenchyme development.

### Twist1 function is required in migratory CNCCs for ectomesenchyme specification

We next investigated whether Twist1 functions in CNCCs for ectomesenchyme formation. To do so, we developed a transgenic strategy to misexpress a dominant-negative Twist1b protein specifically in migratory CNCCs. *Caenorhabditis elegans* has a single Twist gene, *hlh8*, which is required for mesodermal patterning and vulval muscle development [23]. Mutation of Glutamine 29 to Lysine in the DNA-binding domain of Hlh8 has been shown to dominantly interfere with its function [24]. As this residue is conserved in zebrafish Twist1 genes (Glutamine 84 in Twist1b), we reasoned that the equivalent mutation might also result in a dominant-negative protein. We therefore constructed a transgenic line in which Twist1b-E84K (referred to as dnTwist1b) was expressed under the Gal4VP16-sensitive *UAS* promoter. We also constructed a second transgenic line in which the Gal4VP16 transcriptional activator was expressed under the control of a *sox10* neural crest promoter [25]. Although the endogenous *sox10* gene is first expressed in pre-migratory CNCCs at 10.5 hpf, in situ hybridizations for *kikGR* mRNA in *sox10*:Gal4VP16; *UAS*:kikGR embryos revealed transgene expression within migratory CNCCs at 13 hpf, but not earlier within premigratory CNCCs at 11 hpf (Figure S2). Hence, this *sox10*:Gal4VP16 line allows us to alter genetic pathways specifically in migratory CNCCs. Consistent with a requirement for Twist1 in migratory CNCCs, we observed persistent *sox10* and reduced *fli1a* expression in the arches of *sox10*:Gal4VP16; *UAS*:dnTwist1b embryos, similar to what we observed in *twist1a/1b*-MO embryos (Figure 1). We also observed a partial reduction of arch *dlx2a* expression upon dnTwist1b misexpression, as well as reductions of the facial skeleton, particularly in the jaw region (Figure 1K, 1L, 1O, 1P). Hence, the similar phenotypes of *twist1a/1b*-MO embryos and transgenic embryos with CNCC-specific disruption of Twist1 function indicate that zebrafish Twist1 genes function largely in migratory CNCCs for ectomesenchyme development.

### Twist1 genes globally activate ectomesenchyme and inhibit non-ectomesenchyme gene expression

**Figure 2:**
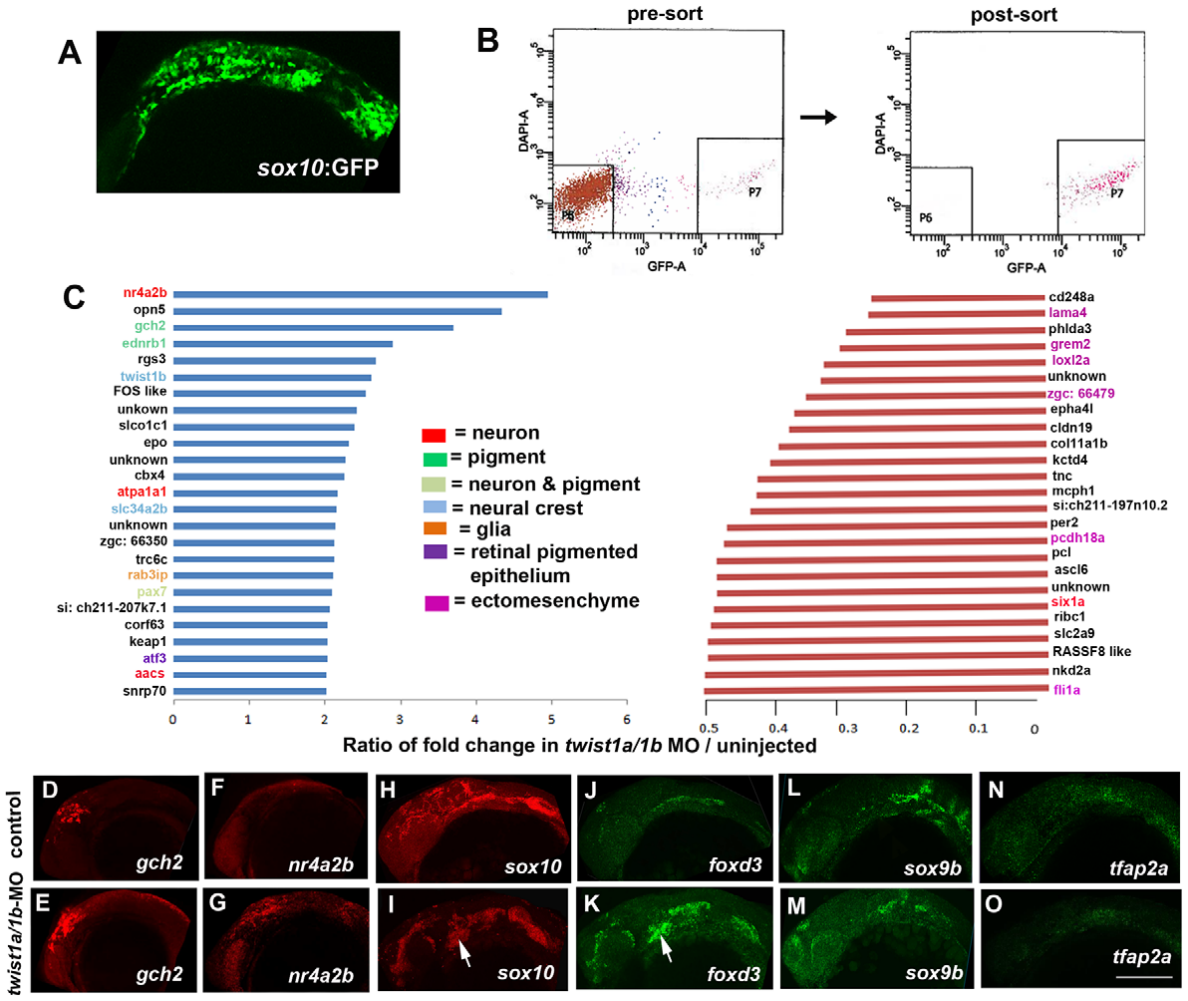
Gene expression profiling in *twist1a/1b*-MO embryos. (A,B) *sox10*:GFP-positive and -negative cells were isolated from un-injected or *twist1a/1b*-MO embryos at 18 hpf by FACS. (C) Fold changes of the GFP+/GFP− ratios between *twist1a/1b*-MO and un-injected controls show the top 25 up-regulated (blue) and down-regulated (red) genes after Twist1 depletion. Color codes indicate where genes are expressed based on the published literature (see Table S1 for references). (D–O) Confocal projections of fluorescent in situ hybridizations show expanded expression of *gch2* and *nr4a2b* at 18 hpf, ectopic arch expression (arrows) of *sox10* and *foxd3* at 24 hpf, but no change in *sox9b* and *tfap2a* expression at 24 hpf in *twist1a/1b*-MO versus un-injected controls. Scale bar = 50 µm.

Twist1 could regulate the transition from early multipotent CNCCs to ectomesenchyme precursors, or alternatively fate choices between ectomesenchyme and non-ectomesenchyme lineages. In order to examine these possibilities without bias, we performed microarray-based gene expression profiling of purified *sox10*:GFP-positive CNCCs from 18 hpf un-injected or *twist1a/1b*-MO-injected embryos (Figure 2A, 2B). We next compared the relative enrichment of transcripts in GFP-positive versus GFP-negative CNCCs of un-injected and *twist1a/1b*-MO embryos to identify CNCC-specific genes up or down regulated by Twist1 depletion (Figure 2C). By comparing the GFP+/GFP− ratio between un-injected and *twist1a/1b*-MO embryos (as opposed to simply comparing expression levels between GFP+ fractions), we controlled for possible non-specific effects of MO toxicity on gene expression (i.e. non-specific toxicity is predicted to equally affect GFP+ and GFP− fractions and thus toxicity effects would cancel out in the ratio). By analyzing data sets with respect to published gene expression patterns (Table S1), we found that the down-regulated set included many known ectomesenchyme genes (e.g. *lama4*, *grem2*, *loxl2a*, *pcdh18a*, and *fli1a*) and the up-regulated set included genes expressed in non-ectomesenchyme lineages such as neurons (e.g. *nr4a2b*, *atpa1a1*, and *aacs*), glia (e.g. *rab3ip*), and pigment (e.g. *gch2* and *ednrb1*) (Figure 2C). In order to validate several of the most differentially expressed genes, we next performed in situ hybridization. Whereas the ectomesenchyme expression of *fli1a* was severely reduced (Figure 1F), the non-ectomesenchyme expression domains of *nr4a2b* and *gch2* were increased in intensity and expanded in size in *twist1a/1b*-MO embryos at 18 hpf (Figure 2D–2G). However, unlike *sox10*, *nr4a2b* and *gch2* were not ectopically expressed in Twist1-depleted arches. As expected, *sox10* was also upregulated 1.28 fold. This modest upregulation is likely due to the more widespread expression of *sox10* in non-ectomesenchyme and trunk neural crest cells not affected by loss of Twist1 function, thus diluting the effect of the specific ectomesenchyme upregulation when total neural crest fractions are analyzed. This limitation indicates that other genes that behave like *sox10* may have been overlooked in our analysis.

Although early CNCC genes did not appear as top hits in our microarray analysis, *sox10* expression did abnormally persist in *twist1a/1b*-MO ectomesenchyme (Figure 2I). We therefore asked whether this was a general property of genes expressed in the early CNCC domain. *sox10*, *sox9b*, *tfap2a*, and *foxd3* are expressed in wild-type CNCCs beginning around 10.5 hpf but not in arch ectomesenchyme at 24 hpf. In Twist1-depleted embryos, we observed ectopic arch expression of *foxd3* but not *sox9b* and *tfap2a* (Figure 2J–2O). Whereas several studies have established roles for Sox10 and Foxd3 in maintaining neural crest multipotency [26], these transcription factors also have later roles in promoting non-ectomesenchyme lineages such as melanocytes, neurons, and glia [27], [28]. The lack of *sox9b* and *tfap2a* upregulation in *twist1a/1b*-MO embryos argues against a general perdurance of early CNCC expression, with the ectopic expression of *sox10* and *foxd3* likely reflecting the upregulation of non-ectomesenchyme gene expression observed in our microarray analysis. However, despite the ectopic expression of *sox10* and *foxd3* in 24 hpf *twist1a/1b*-MO arches, we failed to observe ectopic *foxd3*-positive glia, HuC/D-positive cranial ganglionic neurons, *dct*-positive melanophores, or *xdh*-positive xanthophores in the arches of either *twist1a/1b*-MO or *sox10*:Gal4VP16; *UAS*:dnTwist1b larvae at later stages (Figure 1Q–1AB and Figure S3). Instead, the *sox10*:GFP-positive arches of *twist1a/1b*-MO embryos were reduced at 36 hpf, and even more so at 48 hpf, with Lysotracker staining revealing increased cell death in arch CNCCs and putative CNCCs in the dorsal neural tube (Figure S4 and data not shown). In addition, expression of the early chondrocyte marker *sox9a* was very reduced in arch CNCCs of 48 hpf *twist1a/1b*-MO embryos (Figure S4G) yet was less affected in the mesoderm-derived precursors of the otic capsule cartilage which is less sensitive to loss of Twist1a/1b function (Figure 1N). Hence, arch CNCCs undergo cell death and fail to initiate skeletal gene expression in the absence of Twist1 function yet do not form ectopic non-ectomesenchyme derivatives.

### Twist1 is required for Fgf signaling in ectomesenchyme precursors

**Figure 3:**
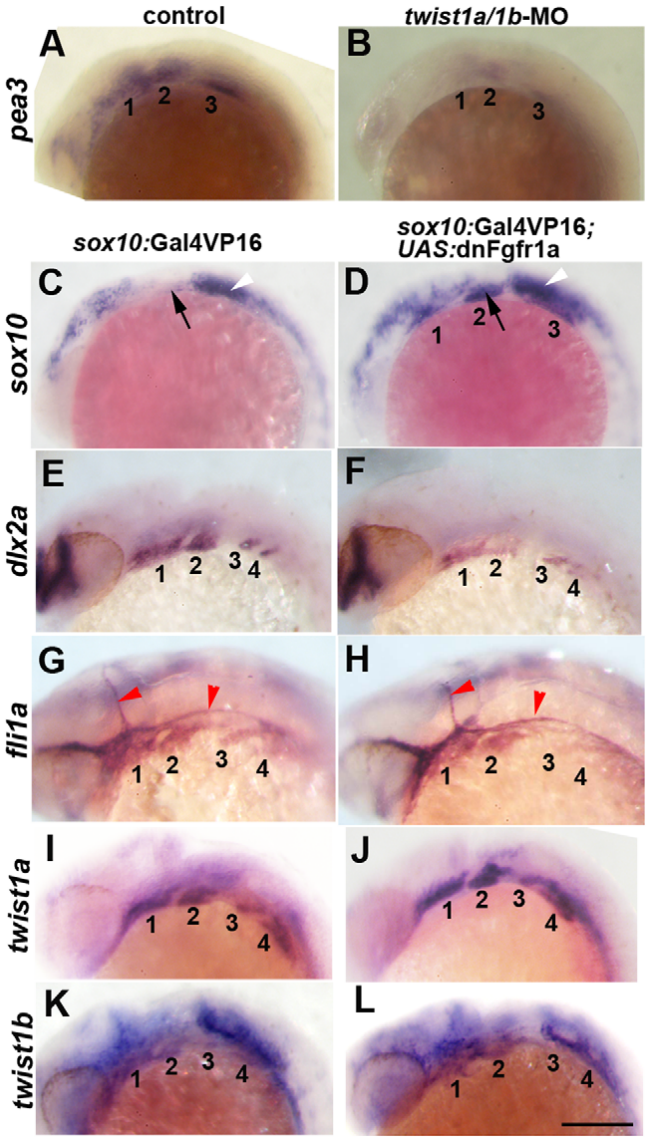
Fgf signaling depends on Twist1 and regulates a subset of ectomesenchyme gene expression. (A,B) In situs at 18 hpf show that expression of the Fgf target gene *pea3* is reduced in the arches (numbered) of *twist1a/1b*-MO embryos (n = 8/8) compared to un-injected controls (n = 0/12). (C–L) *sox10*:Gal4VP16; *UAS*:dnFgfr1a embryos show ectopic arch expression of *sox10* at 18 hpf (n = 8/8) versus controls (n = 0/8), reduction of *dlx2a* at 24 hpf (n = 3/3) versus controls (n = 0/3), but no change in *fli1a* at 24 hpf (n = 0/7) versus controls (n = 0/5). *twist1a* expression was unchanged at 24 hpf in dnFgfr1a embryos (n = 10) compared to controls (n = 7), as was *twist1b* expression in dnFgfr1a embryos (n = 8) compared to controls (n = 8). Black arrows indicate the second arch, white arrowheads the ear, and red arrowheads the vasculature. Scale bar = 50 µm.

As Fgf signaling has also been shown to promote ectomesenchyme fates [18], [19], we next examined whether Twist1 might be required for Fgf signaling during ectomesenchyme development. In zebrafish embryos, *pea3* expression is entirely dependent on Fgf signaling [29], and we observed decreased *pea3* expression in the arches of *twist1a/1b*-MO embryos at 18 hpf (Figure 3B), consistent with Twist1 being required for Fgf signaling in migrating CNCCs. We next investigated to what extent loss of Fgf signaling accounts for the ectomesenchyme gene expression defects seen upon Twist1 depletion. To do so, we inhibited Fgf signaling specifically in migratory CNCCs through transgenic misexpression of a dominant-negative version of Fgfr1a (dnFgfr1a). In *sox10*:Gal4VP16; *UAS*:dnFgfr1a embryos, we observed persistent *sox10* and reduced *dlx2a* expression in the arches, yet, unlike Twist1-deficient embryos, *fli1a* expression was largely unaffected (Figure 3C–3H). This effect of Fgf inhibition on ectomesenchyme formation was not due to transcriptional regulation of Twist1 genes as both *twist1a* and *twist1b* were expressed normally in 24 hpf *sox10*:Gal4VP16; *UAS*:dnFgfr1a embryos (Figure 3I–3L). We therefore conclude that Fgf signaling likely functions downstream of Twist1 to repress *sox10* and activate *dlx2a* expression yet plays less of a role in *fli1a* expression.

### Twist1 regulates *fli1a* expression through an ectomesenchyme-specific enhancer

**Figure 4:**
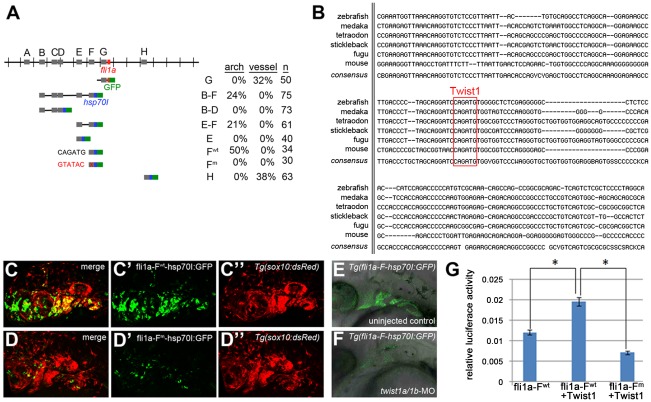
Twist1 regulates *fli1a* expression through an ectomesenchyme-specific enhancer. (A) Schematic shows the *fli1a* genomic locus with hatch marks at 1 kb intervals. Predicted enhancers (grey boxes, “A–H”) and the first exon (red box) are shown. Below are the various enhancer constructs analyzed, with the *hsp70I* core promoter in blue and GFP in green. Wild-type (black) and mutant (red) versions of the putative Twist1 binding site are also shown, as is the percentage of transient transgenic embryos showing GFP expression in the pharyngeal arches or vasculature. (B) Alignment of the central portion of the F enhancer between five fish species and mouse. The putative bHLH/Twist1 binding site is boxed in red. (C,D) Confocal projections of 32 hpf *sox10*:dsRed embryos injected at the one-cell-stage with *fli1a*-F enhancer constructs. The wild-type (C) but not mutant (D) enhancer drives arch expression. (E and F) Confocal sections of merged GFP and DIC channels show that a stable *Tg(fli1a-F-hsp70I:GFP)* line displays arch GFP expression in 32 hpf wild-type (E) but not *twist1a/1b*-MO (F) embryos. (G) Luciferase activity relative to renilla firefly activity in 293T cells transfected with wild-type or mutant *fli1a*-F enhancer reporter constructs, with or without a Twist1 expression plasmid. Asterisks indicate significant comparisons using a student's t-test (p<0.05). Arbitrary units and standard error of the mean are shown.

As we determined that Fgf signaling is not required for *fli1a* ectomesenchyme expression, we next examined whether Twist1 might more directly activate *fli1a* expression. We first used a comparative genomics and transient transgenic approach to identify ectomesenchyme-specific regulatory regions of the *fli1a* gene. A ∼15 kb region centered around the first exon of the *fli1a* gene had previously been shown to drive expression in both the ectomesenchyme and vasculature [30], and we used the mVISTA program [31] to identify seven short (<500 bp) sub-regions (A–H) that were conserved between zebrafish, puffer fishes (*Takifugu rubripes* and *Tetraodon nigroviridis*), stickleback (*Gasterosteus aculeatus*), and medaka (*Oryzias latipes*). By testing the ability of these sub-regions to drive GFP expression in conjunction with the *hsp70I* core promoter, we identified two vasculature-specific enhancers (G and H) and one ectomesenchyme-specific enhancer (F) (Figure 4A). Element F, located ∼1.5 kb upstream of the *fli1a* promoter, was sufficient to drive GFP expression in the ectomesenchyme from 19 hpf onwards, as confirmed by co-localization with a *sox10*:dsRed transgene (Figure 4C). We also identified homologous sequence to enhancer F ∼1.7 kb upstream of the mouse *Fli1* gene, with both the mouse and fish Fli1 genes containing a perfectly conserved element, CAGATG, that matches the bHLH binding site, CANNTG (though Twist1 most strongly prefers CATATG) [32] (Figure 4B). This putative Twist1 binding site was required for enhancer function, as mutation to GTATAC abolished the ability of the *fli1a* element F enhancer to direct ectomesenchyme expression in zebrafish embryos and to potentiate Twist1-dependent transgene expression in mammalian 293T cells (Figure 3D, 3G). Twist1 was also required for enhancer F activity as injection of *twist1a/1b*-MO prevented ectomesenchyme expression in a stable *Tg(fli1a-F-hsp70I:GFP)* transgenic line, though this effect could equally likely be indirect (Figure 4E, 4F). Together, our results are consistent with Twist1 activating *fli1a* expression through a conserved enhancer element.

### Exclusion of *id2a* expression is necessary for ectomesenchyme specification

**Figure 5:**
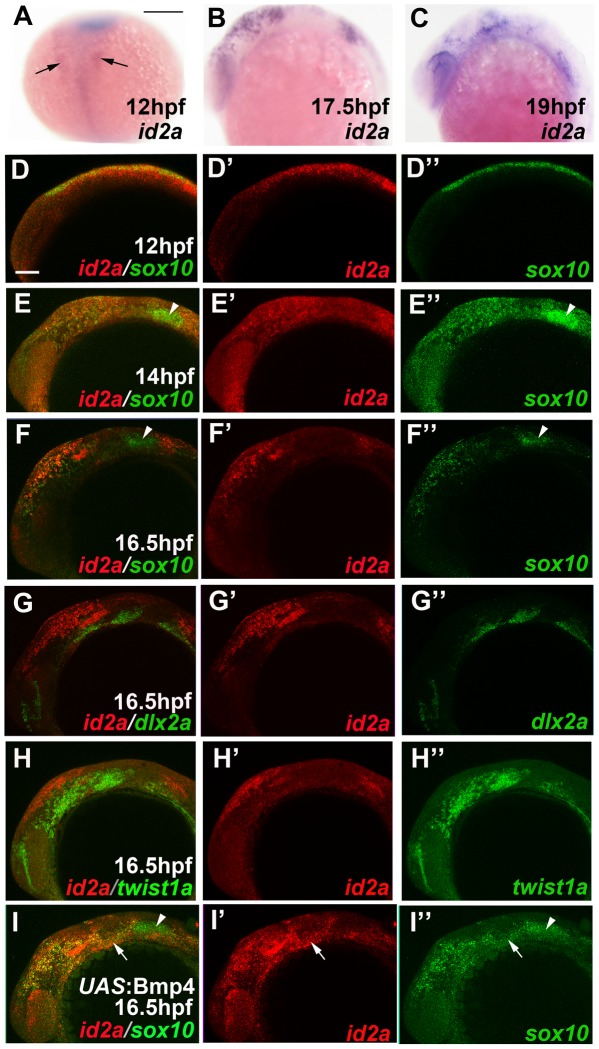
*id2a* is regulated by Bmps and excluded from the ectomesenchyme. (A–C) Colorimetric in situs show weak expression of *id2a* in pre-migratory CNCCs at 12 hpf (dorsal view with anterior up, arrows indicate bilateral CNCC fields) and increasing expression in non-ectomesenchyme precursors at 17.5 and 19 hpf (lateral views). (D–I) Confocal projections of fluorescent in situs show co-localization of *id2a* with *sox10* in CNCCs at 12 hpf (D) and 14 hpf (E), as well as co-localization in the non-ectomesenchyme at 16.5 hpf (F). At 16.5 hpf, *id2a* is excluded from ectomesenchyme CNCCs marked by *dlx2a* (G) and *twist1a* (H), with *twist1a* also being expressed in head mesoderm. In *sox10:*Gal4VP16; *UAS*:Bmp4 embryos, *id2a* and *sox10* are expressed ectopically in the ectomesenchyme (white arrows). Arrowheads indicate otic expression of *sox10*. Scale bars = 50 µm.

**Figure 6:**
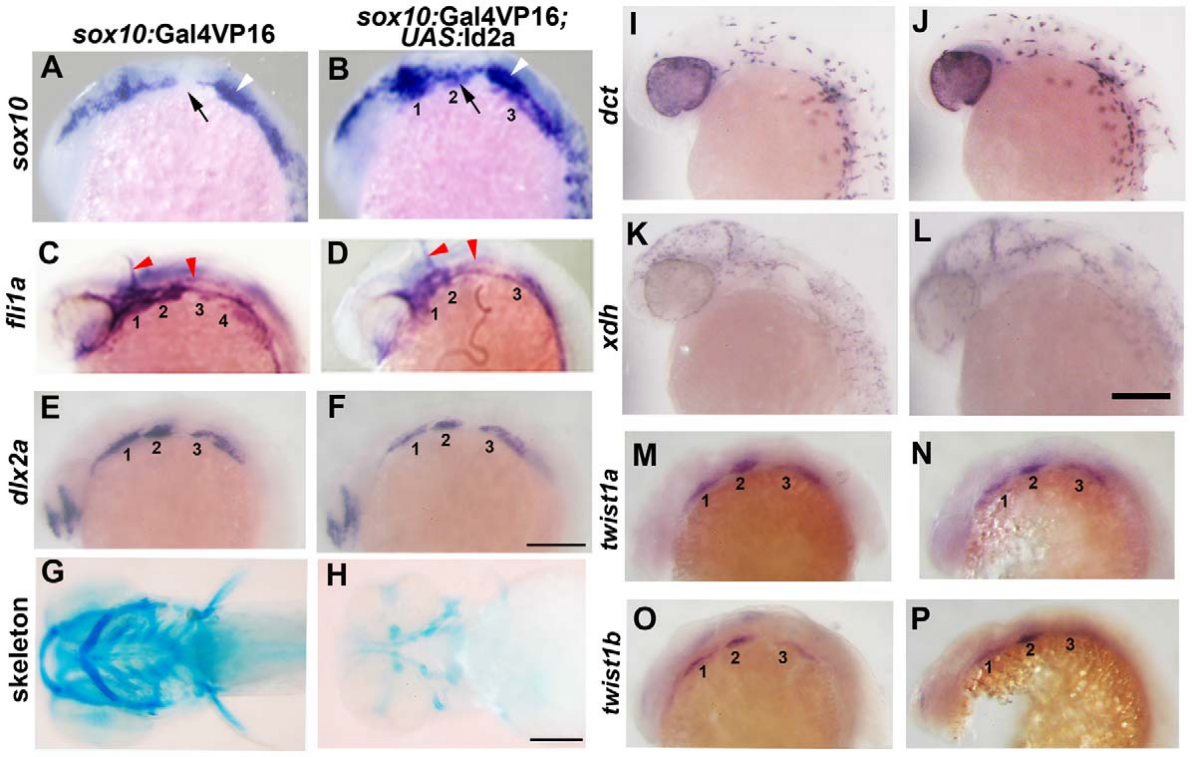
Forced expression of Id2a in CNCCs inhibits ectomesenchyme specification. (A–F) Whole mount in situs show ectopic arch expression of *sox10* at 18 hpf in *sox10*:Gal4VP16; *UAS*:Id2a embryos (n = 7/7) but not *sox10*:Gal4VP16 only controls (n = 0/9), loss of *fli1a* arch expression at 24 hpf in *sox10*:Gal4VP16; *UAS*:Id2a embryos (n = 4/4) but not *sox10*:Gal4VP16 only controls (n = 0/17), and mild reductions of *dlx2a* arch expression at 18 hpf in *sox10*:Gal4VP16; *UAS*:Id2a embryos (n = 5/5) but not *sox10*:Gal4VP16 only controls (n = 0/8). Arches are numbered. Arrows denote the second arch, white arrowheads the developing ear, and red arrowheads the vasculature. (G and H) Skeletal staining at 5 dpf shows severe reduction of the craniofacial skeleton in *sox10*:Gal4VP16; *UAS*:Id2a embryos (n = 17/17) compared to *sox10*:Gal4VP16 only controls (n = 0/22). (I and J) In situs for *dct* expression at 28 hpf show that melanophore precursors are unaffected in *sox10*:Gal4VP16; *UAS*:Id2a embryos (n = 8) and *sox10*:Gal4VP16 only controls (n = 8). (K and L) In situs for *xdh* expression at 28 hpf show that xanthophore precursors are unaffected in *sox10*:Gal4VP16; *UAS*:Id2a embryos (n = 4) and *sox10*:Gal4VP16 only controls (n = 4). (M–P) *twist1a* expression at 18 hpf is unaffected in *sox10*:Gal4VP16; *UAS*:Id2a embryos (n = 12) versus *sox10*:Gal4VP16 only controls (n = 11). *twist1b* expression at 18 hpf is similarly unaffected in *sox10*:Gal4VP16; *UAS*:Id2a embryos (n = 7) versus *sox10*:Gal4VP16 controls (n = 8). Id2a misexpression embryos are to the right in I–P. Scale bars = 50 µm.

As Twist1 genes are expressed throughout the early CNCC domain (e.g. *twist1b* in zebrafish), we next examined whether dimerization partners might regulate the ectomesenchyme-specific function of Twist1. The Id class of proteins modulates Twist1 function by preventing the formation of Twist1-class B heterodimers [14], [15], and several Id family members are expressed in early CNCCs [33], [34], [35]. Intriguingly, we found that zebrafish *id2a* was broadly co-expressed with *sox10* in early CNCCs (12 and 14 hpf) yet by 16.5 hpf was restricted to *sox10*-positive non-ectomesenchyme and excluded from ectomesenchyme expressing *dlx2a* and *twist1a* (Figure 5). As the down-regulation of *id2a* expression tightly correlates with ectomesenchyme specification, we next investigated whether the exclusion of Id2a from ectomesenchyme precursors was required for their differentiation. Indeed, when Id2a was misexpressed throughout migratory CNCCs in *sox10*:Gal4VP16; *UAS*:Id2a embryos, we observed persistent expression of *sox10* and reduced expression of *fli1a* in arch CNCCs, as well as mild reductions of *dlx2a* expression (Figure 6A–6F). The effect of Id2a misexpression was specific to the ectomesenchyme, as we observed severe reductions of the craniofacial skeleton but no defects in the non-ectomesenchyme-derived melanophore and xanthophore precursors (Figure 6G–6L) and HuC/D-positive cranial ganglionic neurons (Figure S3). However, we note that the *sox10* promoter also drives later expression in chondrocytes [36], and thus we cannot rule out that the skeletal defects of *sox10*:Gal4VP16; *UAS:*Id2a animals are additionally or alternatively due to this later phase of Id2a misexpression. In any event, the ectomesenchyme specificity of gene expression and differentiation defects suggests that Id2a does not generally inhibit CNCC differentiation but instead specifically restricts ectomesenchyme fates. Furthermore, the highly similar defects seen upon Id2a misexpression and depletion of Twist1 are consistent with Id2a functioning to inhibit Twist1 in CNCCs. However, this inhibition of Twist1 function by Id2a is likely at the protein and not transcriptional level as *twist1a* and *twist1b* expression were unaffected by Id2a misexpression (Figure 6M–6P).

### Bmp signaling regulates *id2a* expression in non-ectomesenchyme CNCCs

**Figure 7:**
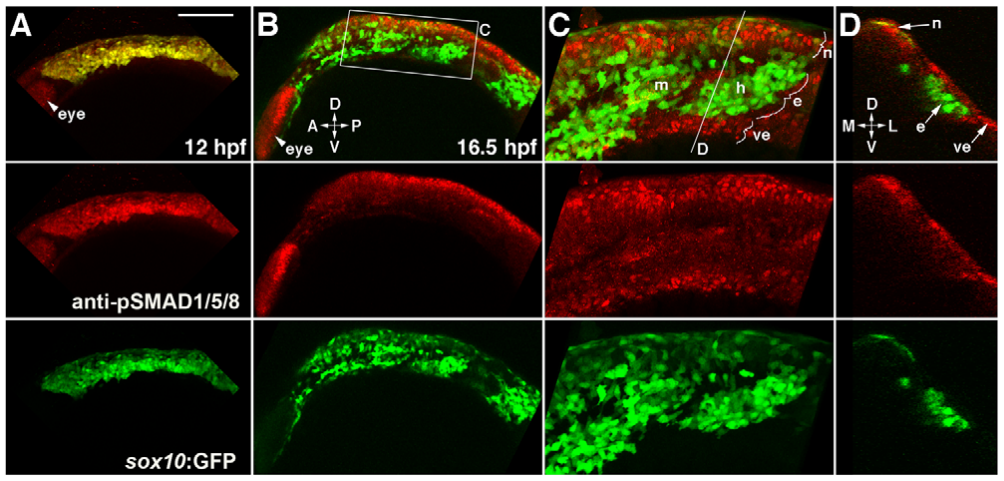
Bmp activity is selectively down-regulated in ectomesenchyme precursors. (A) Confocal projection of pSMAD1/5/8 immunostaining at 12 hpf shows that all *sox10*:GFP-positive CNCCs display high pSMAD1/5/8. (B–D) At 16.5 hpf, a projection (B) and a high-magnification section (C, boxed area in B) show that pSMAD1/5/8 remains high in the dorsal non-ectomesenchyme but is lower in the more ventral ectomesenchyme. An orthogonal section (D, taken at the level of the line in C) shows that high pSMAD1/5/8 CNCCs remain in the neural epithelium whereas low pSMAD1/5/8 CNCCs are positioned more medial and ventral. The eye also displays high pSMAD1/5/8. Abbreviations: D, dorsal; V, ventral; A, anterior; P, posterior; M, medial; L, lateral; m, mandibular arch ectomesenchyme; h, hyoid arch ectomesenchyme; n, non-ectomesenchyme; e, ectomesenchyme; ve, ventral epithelium. Scale bar = 50 µm.

We next investigated the mechanism by which *id2a* expression becomes restricted to non-ectomesenchyme precursors. Bmps are well known regulators of Id gene expression [37], and we found that CNCC-specific misexpression of Bmp4 in *sox10*:Gal4VP16; *UAS*:Bmp4 embryos resulted in ectopic expression of *id2a* in arch ectomesenchyme (Figure 5I). To analyze whether down-regulation of Bmp activity also coincides with loss of *id2a* expression in wild-type ectomesenchyme precursors, we performed immunostaining for the phosphorylated form of Smad1/5/8 (pSmad1/5/8), which is thought to broadly indicate canonical Bmp activity [38]. Consistent with the known role of Bmps in neural crest induction [39], all CNCCs displayed high levels of pSmad1/5/8 at pre-migratory stages (12 hpf) (Figure 7A). In contrast, ectomesenchyme precursors down-regulated pSmad1/5/8 by 16.5 hpf, while more dorsal non-ectomesenchyme precursors continued to display high pSmad1/5/8 (Figure 7B,7C). Cross-sectional views demonstrated that ventral CNCCs with low pSmad1/5/8 were located deep to the ectoderm whereas dorsal CNCCs with high pSmad1/5/8 had yet to delaminate and remained in the neural plate ectoderm (Figure 7D).

### Bmp4 misexpression inhibits ectomesenchyme specification

**Figure 8:**
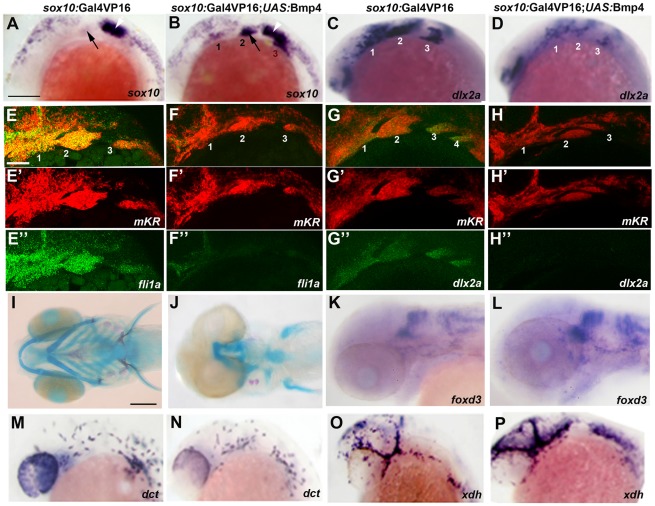
Misexpression of Bmp4 in migrating CNCCs inhibits ectomesenchyme formation. (A–D) Whole mount in situs at 18 hpf show ectopic expression of *sox10* in the arches (numbered) of *sox10*:Gal4VP16; *UAS*:Bmp4; *UAS*:mKR embryos (n = 8/8) compared to *sox10*:Gal4VP16; *UAS*:mKR controls (n = 0/8) and reductions of *dlx2a* in *sox10*:Gal4VP16; *UAS*:Bmp4; *UAS*:mKR embryos (n = 4/4) compared to *sox10*:Gal4VP16; *UAS*:mKR controls (n = 0/4). Arrows indicate the second arch and white arrowheads the developing ear. (E–H) Double fluorescent in situs for *mKR* (red) and *fli1a* or *dlx2a* (green) at 24 hpf show reduction of *fli1a* arch expression in *sox10*:Gal4VP16; *UAS*:Bmp4; *UAS*:mKR embryos (n = 5/5) compared to *sox10*:Gal4VP16; *UAS*:mKR controls (n = 0/9) and reduction of *dlx2a* arch expression in *sox10*:Gal4VP16; *UAS*:Bmp4; *UAS*:mKR embryos (n = 4/4) compared to *sox10*:Gal4VP16; *UAS*:mKR controls (n = 0/3). The mKR transgene was included as a lineage tracer for CNCC-derived cells that migrated into the arches. (I,J) Skeletal staining at 5 dpf shows severe loss of craniofacial skeleton in *sox10*:Gal4VP16; *UAS*:Bmp4; *UAS*:mKR embryos (n = 7/7) compared to *sox10*:Gal4VP16; *UAS*:mKR controls (n = 0/9). (K,L) In situs for *foxd3* at 48 hpf reveal largely normal patterns of glia in *sox10*:Gal4VP16; *UAS*:Bmp4; *UAS*:mKR embryos (n = 8) and *sox10*:Gal4VP16; *UAS*:mKR controls (n = 11). (M–P) In situs at 28 hpf show normal *dct*-positive melanophore precursors in *sox10*:Gal4VP16; *UAS*:Bmp4; *UAS*:mKR embryos (n = 12) and *sox10*:Gal4VP16; *UAS*:mKR controls (n = 15) and normal *xdh*-positive xanthophore precursors in *sox10*:Gal4VP16; *UAS*:Bmp4; *UAS*:mKR embryos (n = 10) and *sox10*:Gal4VP16; *UAS*:mKR controls (n = 10). Scale bars = 50 µm.

We next examined whether the observed down-regulation of Bmp activity in ectomesenchyme precursors was necessary for their formation. Strikingly, Bmp4 misexpression in migratory CNCCs of *sox10*:Gal4VP16; *UAS*:Bmp4 embryos resulted in profound defects in ectomesenchyme specification, including persistent *sox10* expression and reduced expression of *fli1a* and *dlx2a* (Figure 8A–8H). Importantly, the reduction of *fli1a* and *dlx2a* arch expression was not simply due to a lack of CNCC formation, migration, or survival. The examination of *sox10*-positive CNCCs in 15 hpf Bmp4-misexpression embryos revealed no major defects in CNCC induction or migration (Figure S5), and Lysotracker staining also revealed no major increases in CNCC apoptosis (Figure S6). Moreover, although fewer CNCCs were found in the arches of Bmp4-misexpression embryos at later stages (as shown by lineage tracing using the mKR red fluorescent protein at 24 hpf), those CNCCs found in the arches also displayed reduced *fli1a* and *dlx2a* expression (Figure 8E–8H). As with Twist1 depletion or Id2a misexpression, Bmp4 misexpression also resulted in specific reductions of the ectomesenchyme-derived craniofacial skeleton without affecting the non-ectomesenchyme-derived *foxd3*-positive glia, *dct*-positive melanophores, and *xdh*-positive xanthophores (Figure 8I–8P).

### The ectoderm is a likely source of Bmps for ectomesenchyme specification

**Figure 9:**
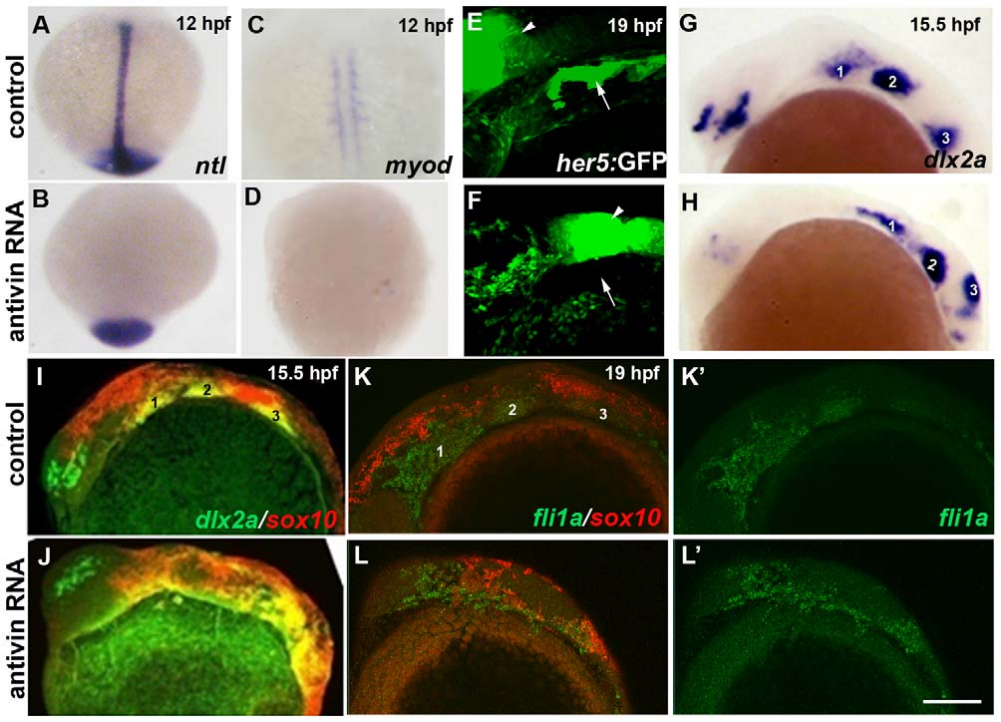
Mesoderm and endoderm are not essential for ectomesenchyme formation. (A–D) In situs at 12 hpf show loss of axial *ntl* and mesodermal *myod* expression in Antivin-mRNA-injected embryos compared to un-injected controls. (E,F) Confocal projections of *her5*:GFP expression at 19 hpf show loss of pouch endoderm (arrows) but not brain (arrowheads) in Antivin-mRNA-injected embryos compared to un-injected controls. (G,H) In situs at 15.5 hpf show normal ectomesenchyme induction of *dlx2a* in Antivin-mRNA-injected embryos (n = 12) and un-injected controls (n = 13). Arches are numbered. (I,J) Double fluorescent in situs at 15.5 hpf show normal induction of *dlx2a* (green) in *sox10*-positive CNCCs (red, yellow indicates co-localization) in Antivin-mRNA-injected embryos (n = 15) and un-injected controls (n = 9). (K,L) Double fluorescent in situs at 19 hpf show complementary expression of *fli1a* (green) in ectomesenchyme and *sox10* (red) in non-ectomesenchyme in Antivin-mRNA-injected embryos (n = 5) and un-injected controls (n = 8). Scale bar = 50 µm.

We also investigated which tissue is the likely source of Bmps for the ectomesenchyme lineage decision. A previous study had reported that ablation of mesoderm and endoderm, by injection of mRNA encoding the Nodal antagonist Antivin, resulted in severe reductions of *dlx2a*-positive ectomesenchyme at 24 hpf [40], yet it was unclear whether the loss of *dlx2a* reflected defects in the specification or later endoderm-mediated survival of ectomesenchyme precursors [41], [42], [43], [44]. By performing similar Antivin mRNA injections, we found normal *dlx2a* and *fli1a* induction at 15.5 hpf and *sox10* down-regulation by 19 hpf in the presumptive ectomesenchyme of embryos lacking endoderm and mesoderm (Figure 9). Hence, rather than signals deriving from the arch endoderm and/or mesoderm [18], our data are more consistent with signals from the non-neural ectoderm, such as Bmps, influencing ectomesenchyme fates.

### Id2a is required for Bmps to inhibit ectomesenchyme formation

**Figure 10:**
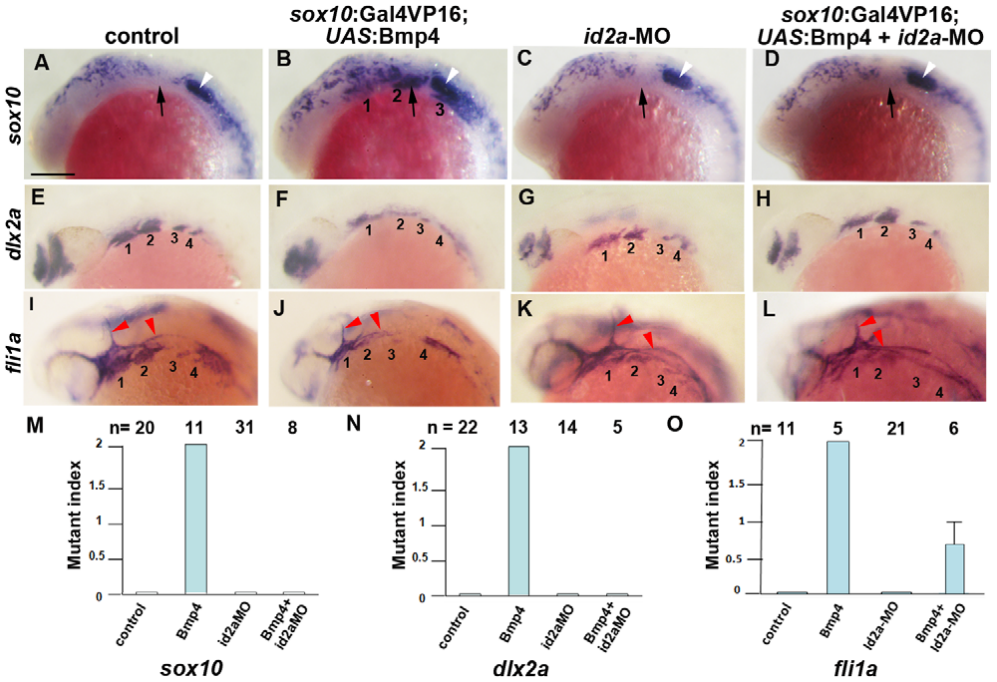
Reduction of Id2a rescues the ectomesenchyme defects caused by Bmp4 misexpression. (A–L) Colorimetric in situs show ectopic *sox10* arch expression at 18 hpf and reductions of *dlx2a* and *fli1a* arch expression at 24 hpf in *sox10*:Gal4VP16; *UAS*:Bmp4 embryos but not *sox10*:Gal4VP16 control or *id2a*-MO-injected embryos. *sox10*:Gal4VP16; *UAS*:Bmp4 embryos injected with *id2a*-MO showed complete rescue of *sox10* and *dlx2a* expression and partial rescue of *fli1a* expression. Arches are numbered and arrows denote the second arch, white arrowheads the developing ear, and red arrowheads the vasculature. Scale bar = 50 µm. (M–O) Quantification of gene expression defects. The mutant index is based on the following: 0 = normal, 1 = partially defective, 2 = fully defective. Fully defective was defined as gene expression being of equal intensity to that seen in un-injected *sox10*:Gal4VP16; *UAS*:Bmp4 embryos. For *sox10*, partially defective was defined as a reduction in the number of expressing cells and/or the intensity of arch expression compared to un-injected *sox10*:Gal4VP16; *UAS*:Bmp4 embryos. For *fli1a* and *dlx2a*, partially defective was defined as a level of arch expression intermediate between un-injected *sox10*:Gal4VP16; *UAS*:Bmp4 and *sox10*:Gal4VP16 control embryos. The rescue of Bmp4 misexpression defects with *id2a*-MO injection was statistically significant for all genes based on a Tukey-Kramer HSD test (α = 0.05). Standard errors of the mean are shown.

As Id2a and Bmp4 misexpression resulted in similar ectomesenchyme defects, we next examined whether Id2a might mediate the negative effects of Bmps on ectomesenchyme development. To do so, we injected a previously validated translation-blocking *id2a*-MO [17] into one-cell-stage embryos to deplete Id2a protein. Whereas injection of *id2a*-MO caused no ectomesenchyme defects on its own, injection into *sox10*:Gal4VP16; *UAS*:Bmp4 embryos fully rescued persistent *sox10* and reduced *dlx2a* arch expression, as well as partially rescuing *fli1a* arch expression (Figure 10). In contrast, injection of *twist1b* mRNA failed to rescue *sox10* persistence in *sox10*:Gal4VP16; *UAS*:Bmp4 embryos (Figure S7). Together, these results support a model in which, rather than inhibiting *twist1a* or *twist1b* at the transcriptional level, Bmps negatively regulate Twist1 ectomesenchyme function through induction of the Id2a negative regulator.

## Discussion

**Figure 11:**
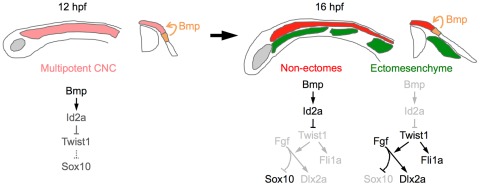
Model of ectomesenchyme specification. At 12 hpf, Bmps in the neural plate border ectoderm promote moderate expression of *id2a* in multipotent CNCCs. As ectomesenchyme precursors delaminate from the neuroepithelium and migrate away from the Bmp source (16 hpf), they downregulate *id2a* expression, which allows Twist1 to induce *fli1a* expression and potentiate Fgf signaling. Fgf signaling then inhibits *sox10* and promotes *dlx2a* expression. In the non-ectomesenchyme, sustained Bmp activity and *id2a* expression dampens Twist1-dependent inhibition of neural, glia, and pigment gene expression programs. Lateral diagrams are shown on the left and cross-sectional diagrams on the right.

Here we present evidence for a new model of ectomesenchyme formation. Upon delamination from the neural plate border, early migrating CNCCs downregulate Bmp activity and *id2a* expression, thus allowing activation of Twist1. Twist1 then promotes ectomesenchyme and inhibits non-ectomesenchyme gene expression through both potentiation of Fgf signaling and activation of genes such as *fli1a* (Figure 11). A salient feature of our model is that the integration of Bmp inhibition at their origin and Fgf activation along their migratory route would confer temporal and spatial specificity to the generation of ectomesenchyme fates from CNCCs.

### Twist1 promotes ectomesenchyme at the expense of non-ectomesenchyme gene expression

The CNCC-derived craniofacial skeleton is nearly completely lost in zebrafish with reduced Twist1a and Twist1b function. This loss of skeleton does not appear to be due to defects in the generation or migration of CNCCs as shown by the presence of largely normal numbers of migrating *dlx2a*-positive CNCCs at 18 hpf and *sox10*:dsRed-positive arch CNCCs at 28 hpf in *twist1a/1b*-MO embryos. Instead, we found that Twist1a and Twist1b are redundantly required for a subset of ectomesenchyme gene expression, in particular the early induction of *fli1a* and inhibition of *sox10*, with the later severe loss of facial skeleton precisely correlating with a loss of pre-chondrogenic *sox9a* expression, as well as increased cell death in the arches. However, *dlx2a* expression was much less affected in *twist1a/1b*-MO embryos, as well as in mice with CNCC-specific deletion of Twist1, suggesting that some aspects of ectomesenchyme formation may be Twist1-independent [11]. Our data in zebrafish are generally in agreement with previous studies of Twist1 function in mouse. Conventional *Twist1^−/−^* mutant mice also displayed arch persistence of *Sox10* and loss of later *Sox9* expression, although *Fli1* and early *Dlx2* expression were not examined and lethality at E10.5–E11.5 precluded an analysis of the extent of craniofacial skeletal loss [12]. In addition, Twist1 function was required largely in CNCCs for *Sox10* repression and craniofacial skeleton development in both zebrafish (this study) and mouse [11], although in both species the facial skeleton was not as affected as in *twist1a/1b*-MO zebrafish. These phenotypic differences might reflect incomplete efficacy of the dnTwist1b transgene at reducing endogenous Twist1 function, or alternatively additional non-autonomous roles of mesodermal Twist1 expression (which is also lost in *twist1a/1b*-MO larvae) in the migration, survival, and/or differentiation of arch CNCCs. Indeed, a role for mesodermal Twist1 in the migration of CNCCs has been inferred from studies in mouse, although we did not observe major defects in CNCC migration in *twist1a/1b*-MO zebrafish.

In theory, Twist1 could promote the transition from a multipotent CNCC precursor to a more lineage-restricted ectomesenchyme cell, or alternatively influence fate choices between ectomesenchyme and non-ectomesenchyme lineages. However, the observation that only a subset of early CNCC genes persisted in *twist1a/1b*-MO arches, combined with the normal development of CNCC-derived cranial pigment and glial cells, suggest that CNCCs do not remain as multipotent precursors in the absence of Twist1 function. Instead, the ectopic arch expression of *sox10* and *foxd3* in Twist1-depleted embryos, at a stage when these genes are normally co-expressed in the non-ectomesenchyme, suggests that presumptive ectomesenchyme precursors partially adopt a non-ectomesenchyme expression profile in the absence of Twist1. This conclusion was confirmed by our microarray analysis in which non-ectomesenchyme but not early CNCC genes were the most upregulated in Twist1-deficient embryos. Unexpectedly, some non-ectomesenchyme genes, such as *nr4a2b* and *gch2*, were upregulated in the non-ectomesenchyme domain but not ectopically expressed in Twist1-deficient arches, suggesting that Twist1 may have additional roles in restricting non-ectomesenchyme gene expression within the early non-ectomesenchyme domain. Consistent with only a subset of non-ectomesenchyme genes showing ectopic arch expression, we also found no evidence for pigment cells, functional glia, or neurons forming ectopically in Twist1-deficient arches. Hence, loss of Twist1 function is not sufficient for arch CNCCs to generate non-ectomesenchyme derivatives.

### Twist1 promotes ectomesenchyme through both Fgf-dependent and -independent gene activation

Our data also indicate that Twist1 promotes ectomesenchyme fates through two largely parallel pathways. The finding that Twist1 was required for expression of the Fgf target gene *pea3* in arch ectomesenchyme suggests a role for Twist1 in potentiating Fgf signaling. Twist1 might do so by regulating expression of Fgf ligands and/or receptors, as Twist1 has been shown to regulate the CNCC expression of both *Fgf10* and *Fgfr1* in mice [12]. Hence, Twist1 could function upstream of autocrine/paracrine Fgf signaling within CNCCs that promotes ectomesenchyme development. However, although Fgf signaling was required to repress *sox10* and activate *dlx2a* expression, we found that *fli1a* ectomesenchyme expression was Fgf-independent. Instead, *fli1a* appears to be a potentially direct target of Twist1, and we have identified a conserved ectomesenchyme enhancer element of *fli1a* that is Twist1-dependent. However, chromatin immunoprecipitation experiments (currently not feasible due to the lack of a high quality Twist1 antibody) will eventually be needed to show enhancer occupancy by Twist1. Moreover, the function of Fli1a in ectomesenchyme formation also remains to be investigated. Targeted disruption of the *Fli1* gene in mouse results in embryonic lethality by E12.5 due to severe hemorrhaging, thus precluding an analysis of its role in development of the ectomesenchyme-derived craniofacial skeleton [45]. In the future, the generation of zebrafish *fli1a* mutants and/or conditional *Fli1* mouse knockouts will allow us to determine the extent to which Fli1 mediates that effects of Twist1 in craniofacial development.

### Id2a restricts Twist1 activity to the ectomesenchyme

The expression of Twist1 genes throughout early CNCCs in both zebrafish [13] and mice [46], [47] suggests that Twist1 function is regulated at the post-translational level during ectomesenchyme formation, either through enzymatic modifications or changes in dimerization partners. Here we find evidence for the latter, with the Bmp target gene Id2a functioning oppositely to Twist1 for ectomesenchyme specification. Previous work suggests that Id2a does not inhibit Twist1 function per se, but instead would modulate what types of dimers Twist1 forms [14], [15]. In the future, the identification of binding partners for Twist1 in CNCCs will allow us to test what roles Twist1 heterodimers versus homodimers play in ectomesenchyme specification. Furthermore, we note that Twist1 genes continue to be expressed in arch ectomesenchyme and likely have additional roles in facial skeletal patterning, for example by interacting with other bHLH factors such as Hand2 [48]. Hence, different Twist1 heterodimers may have distinct roles during craniofacial development (i.e. ectomesenchyme formation versus arch patterning), with the later disruption of these heterodimers in Twist1-deficient and Id2a-misexpression embryos also contributing to some aspects of the observed facial skeletal defects.

### A Bmp-dependent switch for ectomesenchyme formation

Previous studies have shown both temporal and spatial components to the specification of the large diversity of neural crest derivatives. In the trunk, neural crest cell fate is influenced both by migration pathways [49], [50] and signals at their destinations [51]. In contrast, CNCCs appear to be largely fate-restricted before migration in zebrafish, with Wnts from the dorsal ectoderm promoting pigment at the expense of neural and glial fates but having little effect on ectomesenchyme derivatives [4], [5]. Instead, we find that Bmps, also from the ectoderm, actively inhibit ectomesenchyme fates in more dorsal CNCCs. Furthermore, this observed role of Bmps in regulating ectomesenchyme specification is not simply an indirect effect of earlier roles of Bmps in neural crest development, as prolonging Bmp activity did not result in decreased amounts of CNCC being generated or defects in non-ectomesenchyme derivatives

The down-regulation of Bmp activity in ectomesenchyme precursors could reflect CNCCs distancing themselves from a Bmp source in the non-neural ectoderm (e.g. Bmp2b) and/or active inhibition of Bmp signaling during migration. Whereas the Bmp antagonist Noggin1 is expressed in the paraxial mesoderm surrounding migratory CNCCs in zebrafish [52], genetic ablation of endoderm and mesoderm did not disrupt ectomesenchyme gene expression, although there are likely other sources of Bmp inhibitors [53]. Alternatively, the down-regulation of Bmp activity could result from the delamination of CNCCs from the neural plate ectoderm, with the basement membrane of the ectodermal epithelium, under which CNCCs migrate, serving as a barrier to apical Bmp secretion and signaling [54].

Although we found that Bmp down-regulation was essential for ectomesenchyme formation, transgenic inhibition of Bmp (or Id2a depletion) did not result in ectopic ectomesenchyme (data not shown). Therefore, low Bmp activity is not sufficient to specify ectomesenchyme fates. Instead, Bmp down-regulation might allow CNCCs to respond to ectomesenchyme-promoting signals present only in the early CNCC milieu. In particular, our data suggest that activation of Twist1 at low Bmp levels allows migrating CNCCs to respond to Fgfs. Although the exact source of Fgfs remains to be determined, we propose that a combination of low Bmp and high Fgf signaling uniquely present in early migrating CNCCs generates ectomesenchyme fates.

## Materials and Methods

### Ethics statement

All animals were handled in strict accordance with good animal practice as defined by the relevant national and/or local animal welfare bodies, and all animal work was approved by the University of Southern California Institutional Animal Care and Use Committee.

### Zebrafish lines and transgenic constructs

Zebrafish were staged as described [55]. Previously reported lines include *Tg(hsp70I:Gal4)^kca4^* [56], *Tg(∼3.4her5:EGFP)^ne1911^* [57], and *Tg(UAS:Bmp4; cmlc2:GFP)^el49^* [53]. *Tg(sox10:Gal4VP16)^el159^*, *Tg(UAS:mKR; cmlc2:GFP)^el15^*, *Tg(UAS:kikGR; α-crystallin:Cerulean)^el377^*, *Tg(UAS:Id2a; α-crystallin:Cerulean)^el405^*, *Tg(UAS:dnFgfr1a; cmlc2:GFP)^el28^*, *Tg(UAS:dnTwist1b; UAS:mcherryCAAX; cmlc2:GFP)^el179^*, *Tg(sox10:dsRED)^el10^, Tg (sox10:LOX-GFP-LOX-hDLX3)^el8^*, and *Tg(fli1a-F-hsp70I:GFP)^el411^* lines were generated using Gateway cloning (Invitrogen) and the Tol2 kit as described [58]. *sox10*:Gal4VP16 was created by combining p5E-*sox10*, pME-Gal4VP16, p3E-IRES-GFP-pA, and pDestTol2pA2. The IRES-GFP was later found to be non-functional in stable transgenics. *UAS:*mKR, *UAS:*kikGR, and *UAS*:dnFgfr1a were generated by combining p5E-*UAS*; pME-mKR, pME-kikGR, or pME-dnFgfr1a; p3E-polyA; and pDestTol2CG2. *UAS*:dnTwist1b was made by combining p5E-*UAS*, pME-dnTwist1b, p3E-*UAS*:mCherryCAAXpA, and pDestTol2CG2. The dnTwist1b clone was made by first amplifying the full-length zebrafish *twist1b* cDNA with primers rTwist1bikL: 5′-GGGGACAAGTTTGTACAAAAAAGCAGGCTCGGCCACCATGCCCGAAGAGCCCGCGGAGA-3′ and Twrk: 5′-GGGGACCACTTTGTACAAGAAAGCTGGGTACTTAGATGCAGACATGGACCAAGCGCC-3′. PCR based mutagenesis was then performed with primers twdnEK1L: 5′-GCGCTTTCGCAGAGACTTA-3′ and twdnEK1R 5′-CTGTCGCTTGCGCACGTT-3′ as described [59] to change nucleotide G250 of the *twist1b* cDNA to A, which results in conversion of amino acid 84E to K, with the PCR product cloned into pDONR221 to create pME-dnTwist1b. To construct pME-dnFgfr1a, we amplified a truncated form of zebrafish *fgfr1a* based on the previously reported dominant-negative Fgf receptor in *Xenopus laevis* [60] and cloned the PCR product into pDONR221; primers used were 5′-GGGGACAAGTTTGTACAAAAAAGCAGGCTCCACCATGATAATGAAGACCACGCTG-3′ and 5′GGGGACCACTTTGTACAAGAAAGCTGGGTCTAAGAGCTGTGCATTTTGGC-3′. Full-length zebrafish *id2a* was amplified with primers id2a-F: 5′-GGGGACAAGTTTGTACAAAAAAGCAGGCTCGGCCACCATGAAGGCAATAAG-3′ and id2a-R: 5′-GGGGACCACTTTGTACAAGAAAGCTGGGTGTTAACGGTAAAGTGTCCT-3′ and then cloned into pDONR221 to generate pME-Id2a. *UAS*:Id2a was generated by combining p5E-UAS, pME-Id2a, p3E-polyA, and pDestTol2AB2. *sox10*:dsRED and *sox10*:LOX-GFP-LOX-Dlx3 were generated by combining p5E-*sox10*, p3E-polyA, and pDestTol2pA2 with either pME-dsRed or pME-LOX-GFP-LOX-Dlx3, respectively. *UAS*:mKR was made by combining p5E-*UAS*, pME-mKR, p3E-polyA, and pDestTol2CG2. pME-mKR was made by PCR amplification of membraneKillerRed (mKR) with primers kilredL: 5′-GGGGACAAGTTTGTACAAAAAAGCAGGCTCGGTCGCCACCATGCTGTG-3′ and kilredR: 5′-GGGGACCACTTTGTACAAGAAAGCTGGGTTTAATCCTCGTCGCTACCGA-3′ and insertion into pDONR221. *UAS*:kikGR was made by combining p5E-*UAS*, pME-kikGR, p3E-polyA, and pDestTol2AB2. *fli1a* enhancers were amplified with the following primer sets from zebrafish genomic DNA and cloned into the pDONR-p4p1R vector: (G) Fli-2: 5′-GGGGACAACTTTGTATAGAAAAGTTGTAGAGCGTGCTCGGTAACTG-3′ and Fli-3: 5′-GGGGACTGCTTTTTTGTACAAACTTGGAGCCCGACAATATTCCAAA-3′, (B–F) Fli-6: 5′-GGGGACAACTTTGTATAGAAAAGTTGTTGCACTGGGCAAACTTTAG-3′ and Fli-15: 5′-GGGGACTGCTTTTTTGTACAAACTTGCTCTAGACGCTCAGCCAACC-3′, (B–D) Fli-6 and Fli-13: 5′-GGGGACTGCTTTTTTGTACAAACTTGAGGCTGTCCTGACTGCTGAT-3′, (E–F) Fli-14: 5′-GGGGACAACTTTGTATAGAAAAGTTGAGCACTTCAGGGTTTTTCCA-3′ and Fli-15, (E) Fli-14 and Fli-18: 5′-GGGGACTGCTTTTTTGTACAAACTTGATTGCGTCCAGTTAGATCGG-3′, (F) Fli-19: 5′-GGGGACAACTTTGTATAGAAAAGTTGATGTTGCATCACTTTTCCCC-3′ and Fli-15, and (H) Fli-16: 5′-GGGGACAACTTTGTATAGAAAAGTTGATGGTTTGTTGCAGTCGGTT-3′ and Fli-17: 5′-GGGGACTGCTTTTTTGTACAAACTTGCGCAGGATTACGCTGGAATA-3′. pME-*hsp70I*:GFP was constructed by fusion PCR of the *hsp70I* promoter and full-length GFP. PCR-based mutagenesis of the *fli1a-F* enhancer was performed using primers: 5′-GTATACTGGGGCTCTCGAGG-3′ and 5′-CTAGCAGGATCGTATACTGGG-3′. p5E enhancer vectors were then combined with pME-*hsp70I*:GFP, p3E-polyA, and pDestTol2pA2. To construct stable transgenics, one-cell-stage embryos were injected with vectors and transposase RNA and multiple stable lines were identified for each transgene: *Tg(sox10:Gal4VP16)* (1), *Tg(UAS:Id2a; α-crystallin:Cerulean)* (5), *Tg(UAS:dnTwist1b; UAS:mcherryCAAX; cmlc2:GFP)* (7), *Tg(UAS:dnFgfr1a; cmlc2:GFP)* (6), *Tg(UAS:mKR; cmlc2:GFP)* (2), *Tg(UAS:kikGR; cmlc2:GFP)* (3), *Tg(sox10:dsRED)* (1), *Tg(sox10:LOX-GFP-LOX-hDLX3)* (2), and *Tg(fli1-F-hsp70I:GFP)* (2). In the absence of CRE, *Tg(sox10:LOX-GFP-LOX-hDLX3)^el8^* (referred to as *sox10*:GFP in the Results) drives similar GFP expression as the previously reported *Tg(∼4725sox10:GFP)^ba2^* line but without neural crest cell toxicity [25]. For *sox10*:Gal4VP16/*UAS*:transgene experiments, genotyping for Gal4 and *cmlc2* [61] confirmed the observed phenotypes, except for *sox10*:Gal4VP16/*UAS*:Id2a experiments where genotyping for Id2a was performed with primers id2aF: 5′-AGAACACCCCTGACAACACT-3′ and id2aR: 5′- GCTAATACGACTCACTATAGGTACCGGCAGTCCAATTTC-3′.

### In situ hybridization, immunohistochemistry, and skeletal analysis

Alcian Blue staining of cartilage and Alizarin Red staining of bone at 5 dpf, as well as in situ hybridizations, were performed as described [61]. Reported probes include *sox9a* and *sox9b* [62], *tfap2a* [63], and *fli1a* [9]. *id2a*, *sox10*, *fli1a*, *twist1a*, *ntla*, *dct*, *xdh*, *pea3*, *kikGR*, *mKR*, *gch2*, *nr4a2b*, and *foxd3* probes were synthesized with T7 RNA polymerase from PCR products amplified from multistage zebrafish cDNA, with the exception of *id2a* which was amplified from an *id2a* cDNA plasmid (Zebrafish International Resource Center). We used the following primer pairs: *id2a*: id2aF and id2aR, *twist1a*: 5′-CAGAGTCTCCGGTGGACAGT-3′ and 5′-GCTAATACGACTCACTATAGGGTCTTTTCCTGCAGCGAGTC-3′, *ntla*: 5′- GACCACAAGGAAGTCCCAGA-3′ and 5′- GCTAATACGACTCACTATAGGCATTGAGGAGGGAGAGGACA-3′, *dct*: 5′- CGTACTGGAACTTTGCGACA-3′ and 5′-GCTAATACGACTCACTATAGGACCAACACGATCAACAGCAG-3′, *xdh*: 5′-TGAACACTCTGACGCACCTC-3′ and 5′-GCTAATACGACTCACTATAGGTGTTGAAGCTCCAGCAACAC-3′, *foxd3*: 5′-CGGCATTGGGAATCCATA-3′ and 5′-GCTAATACGACTCACTATAGGCAACGAAATGAAATAGAAAGAAGGA-3′, *pea3*: 5′-CCCATATGATGGTCAAACAGG-3′ and 5′- GCTAATACGACTCACTATAGGATTGTCGGGAAAAGCCAAG-3′, *kikGR*: 5′-GAAGATCGAGCTGAGGATGG-3′ and 5′- GCTAATACGACTCACTATAGGGCTCGTACAGCTTCACCTTGT-3′, *mkr*: 5′-GGGCAGAAGTTCACCATCG-3′ and 5′-GCTAATACGACTCACTATAGGGTGGTGAAGCCGATGAAGG-3′, *nr4a2b*: 5′-CCAGGCTCAGTATGGGACAT-3′ and 5′-GCTAATACGACTCACTATAGGTATGTGACGTCGCCAGGTAG-3′, *sox10*: 5′-TGCATTACAAGAGCCTGCAC-3′ and 5′- GCTAATACGACTCACTATAGGAGGAGAAGGCGGAGTAGAGG-3′, and *gch2*: 5′- GGAATACCAAAAGGCAGCAG-3′ and 5′-GCTAATACGACTCACTATAGGCTCTCTGAACACACCCAGCA-3′. Immunostaining was performed as described [53] using the following antibodies: rabbit anti-pSMAD1/5/8 (Cell Signaling, #9511S, 1∶1000), goat anti-rabbit Alexa-568 (Molecular Probes, 1∶1000), mouse anti-HuC/D (1∶100; Molecular Probes), and goat anti-mouse Alexa-488 (Molecular Probes 1∶1000). Cell death assays with Lysotracker Red were performed as described [53].

### Morpholino and mRNA injections

One-cell-stage embryos were injected with 3 nl of the following translation-blocking MOs (GeneTools, Philomath, OR, USA): *twist1a*-MO 5′- ACCTCTGGAAAAGCTCAGATTGCGG-3′ (400 µM), *twist1b*–MO 5′-TTAAGTCTCTGCTGAAAGCGCGTG-3′ (400 µM), *twist1a/1b*-MO (200/200 µM), and *id2a*-MO 5′-GCCTTCATGTTGACAGCAGGATTTC-3′ (800 µM) [17]. For MO validation, we performed rescue experiments with in vitro transcribed mRNA generated using the Ambion Message machine kit (Applied Biosystems) from a CMV/SP6-Twist1b template generated by combining p5E-CMV/SP6, pME-Twist1b, p3E-polyA, and pDestTol2pA2. Antivin mRNA was synthesized and injected as described [40].

### Imaging

Larval skeletons and colorimetric in situ hybridization embryos were imaged using a Leica MZ16F dissecting microscope using Camera Window software for a Canon60 Shot 80S digital camera. Fluorescent images were captured using a Zeiss LSM5 confocal microscope with Zen software. Identical gain settings were used to acquire fluorescent in situ images across data sets. Images were processed using Photoshop CS4 with care taken to apply identical adjustments throughout data sets.

### Microarray analysis

*sox10*:GFP embryos were injected with *twist1a/1b*-MO. Control and injected embryos were sorted for GFP under a fluorescent dissecting microscope and samples were prepared for FACS using the “cell disruption for flow sorting” protocol described previously [64]. A BD Aria Sorter was used to isolate equal numbers of GFP-positive and GFP-negative cells and RNA was isolated using Trizol (Invitrogen). cDNA was then generated using the WTA2 kit (Sigma Aldrich) and hybridized to a 385K zebrafish array (Roche #05543916001). Microarray data were normalized by Nimblegen, and processed data files were analyzed using Arraystar 4.0 software (DNAstar). Three independent experiments were performed, and a cut-off value of p<0.05 was used for significance. All data was submitted to GEO (Accession #GSE32308).

### Luciferase assay

The Twist1 expression vector was made by cloning the zebrafish *twist1b* cDNA into pCDNA3. Luciferase reporter constructs were generated by cloning wild-type and mutant versions of the *fli1a-F* enhancer into the Pgl3 basic vector (Progema #E1715). We then transfected 293T cells with the Twist1b expression and luciferase reporter plasmids and used the Dual Luciferase Assay kit (Promega #E1910) per manufacturer instructions to measure firefly luciferase activity relative to renilla luciferase activity 48 hours after transfection. All experiments were performed in triplicate.

### Statistical analysis

Statistics were performed with JMP7 software. One-way analysis of variance was calculated with a Tukey-Kramer HSD test (alpha = 0.05) and standard errors of the mean were plotted.

## Supporting Information

Figure S1 Twist1a and Twist1b function redundantly to specify ectomesenchyme. (A–C) In situs at 18 hpf show *sox10* expression in un-injected, *twist1a*-MO, and *twist1b*-MO embryos. A few ectopic *sox10*-positive cells are seen in the second arches (arrows) of *twist1a*-MO and *twist1b*-MO embryos. White arrowheads denote the developing ear. (D–G) Confocal projections of *fli1a*:GFP; *sox10*:dsRed doubly transgenic embryos at 28 hpf show normal *fli1a*:GFP expression in un-injected control, *twist1a*-MO, and *twist1b*-MO embryos and loss of *fli1a*:GFP arch expression in *twist1a/1b*-MO embryos. Arrows indicate *fli1a*:GFP vascular expression which is unaffected in *twist1a/1b*-MO embryos. (H–L) Skeletal staining shows malformed mandibular and hyoid skeletons in *twist1a*-MO and *twist1b*-MO embryos compared to un-injected controls. In addition, co-injection of a Twist1b mRNA not targeted by the MOs partially rescued the head skeleton of *twist1a/1b*-MO embryos (n = 24/24), whereas co-injection of a control *kikGR* mRNA never rescued (n = 0/11). Scale bars = 50 µm.
(TIF)

Figure S2 Time-course of *sox10*:Gal4VP16-dependent transgene expression. (A–D) In situs for *kikGR* mRNA in *sox10:*Gal4VP16; *UAS*:kikGR embryos show transgene expression in migratory CNCCs at 13 hpf but not in pre-migratory CNCCs at 11 hpf. *sox10:*Gal4VP16 control embryos show no expression.
(TIF)

Figure S3 Cranial ganglionic neurons are unaffected in *twist1a/1b*-MO and Id2a misexpression embryos. (A–D) Confocal projections of anti-HuC/D immunofluorescence show neurons of the trigeminal (tg), anterior lateral line (all), otic (ot), and posterior lateral line (pll) ganglia at 36 hpf. No major differences in the pattern of anti-HuC/D staining was observed between uninjected control (n = 6), *twist1a/1b*-MO (n = 6), *sox10*:Gal4VP16 only control (n = 9), and *sox10*:Gal4VP16: *UAS*:Id2a (n = 8) embryos. Scale bar = 50 µm.
(TIF)

Figure S4 Cell death and *sox9a* expression in *twist1a/1b*-MO embryos. (A–D) Confocal projections of Lysotracker Red staining in 36 hpf *sox10*:GFP transgenic embryos show increased cell death in the pharyngeal arches (shown in high-magnification views in C and D from the boxes in A and B) in *twist1a/1b*-MO-injected embryos (n = 6) compared to un-injected controls (n = 6). Arrowhead shows increased cell death in more dorsal CNCCs as well. Scale bar = 50 µm. (E) Quantification of Lysotracker-positive cells per arch area. Mandibular and hyoid arches were used for the analysis. Asterisk indicates statistical significance using a Tukey-Kramer HSD test (α = 0.05). (F,G) In situ hybridizations for *sox9a* at 48 hpf show very reduced expression in the pharyngeal arches of *twist1a/1b*-MO embryos (n = 5/5) compared to uninjected controls (n = 0/12). Dorsal (D) and ventral (V) pre-chondrogenic domains of the mandibular (1) and hyoid (2) arches are shown for the control. *sox9a* expression in the pre-chondrogenic domain of the mesoderm-derived otic capsule cartilage (arrows) was less affected in *twist1a/1b*-MO embryos.
(TIF)

Figure S5 Induction and early migration of CNCCs is unaffected by Bmp4 misexpression. In situs for *sox10* at 15 hpf show no difference in early migrating CNCCs between *sox10*:Gal4VP16; *UAS*:Bmp4 embryos (n = 5) and *sox10*:Gal4VP16 only controls (n = 12). Scale bar = 50 µm.
(TIF)

Figure S6 Cell death analysis in *sox10*:Gal4VP16; *UAS*:Bmp4 embryos. Compared to *sox10*:Gal4VP16 only controls (n = 8), Lysotracker staining reveals no major increase in cell death at 24 hpf in *sox10*:Gal4VP16; *UAS*:Bmp4 embryos (n = 8). Scale bar = 50 µm.
(TIF)

Figure S7 Injection of *twist1b* mRNA fails to rescue *sox10* ectomesenchyme persistence in Bmp4 misexpression embryos. (A–C) *sox10* expression at 18 hpf was similarly upregulated in the arches of uninjected *sox10*:Gal4VP16; *UAS*:Bmp4 embryos (n = 11/11) and *twist1b*-mRNA-injected *sox10*:Gal4VP16; *UAS*:Bmp4 embryos (n = 5/5) compared to *sox10*:Gal4VP16 only controls (n = 0/10). Scale bar = 50 µm.

Table S1 Summary of CNCC genes affected by Twist1 depletion in the microarray analysis.
(XLS)
