# Supplementary figures and images for: Bmps and Id2a Act Upstream of Twist1 To Restrict Ectomesenchyme Potential of the Cranial Neural Crest
Source: PLoS Genet. 2012 May 10;8(5):e1002710. doi: 10.1371/journal.pgen.1002710 (PMC3349740; doi:10.1371/journal.pgen.1002710)

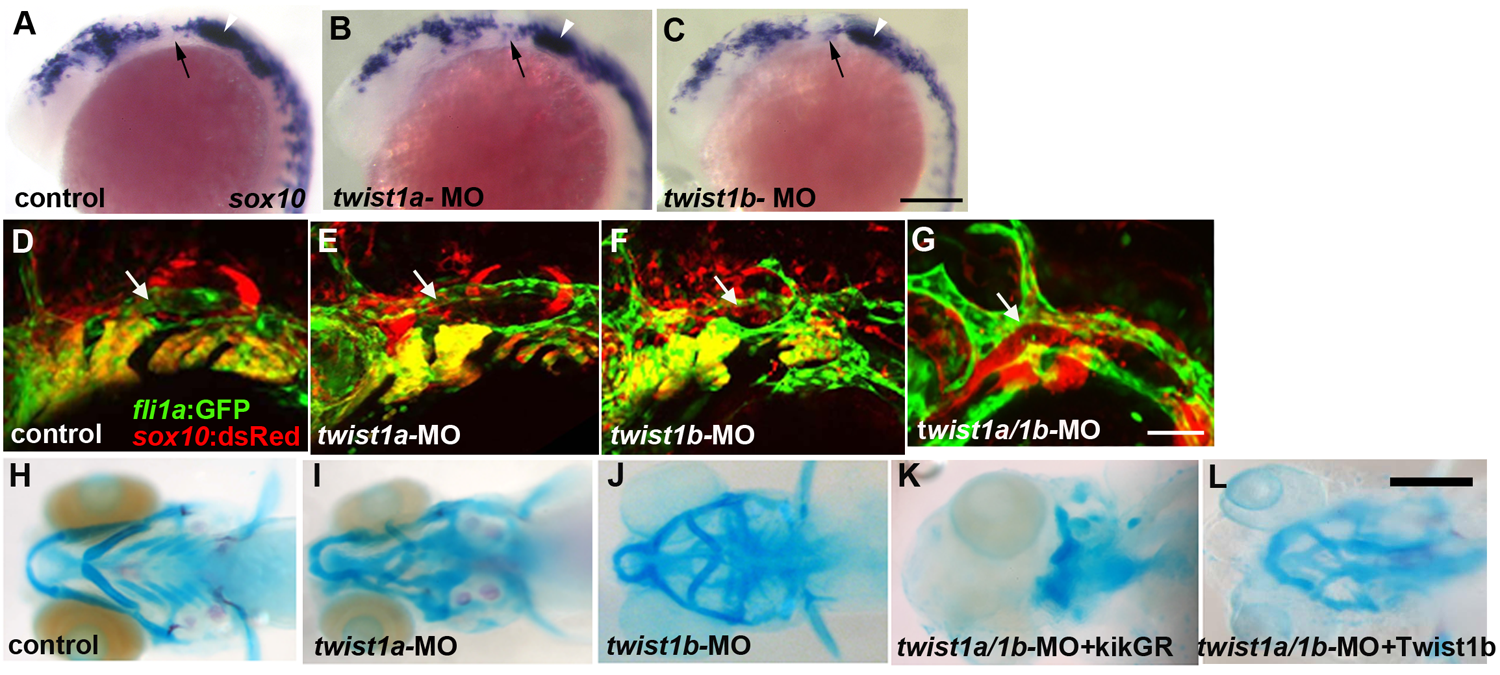

Supplement: Figure S1 — Twist1a and Twist1b function redundantly to specify ectomesenchyme. (A–C) In situs at 18 hpf show sox10 expression in un-injected, twist1a-MO, and twist1b-MO embryos. A few ectopic sox10-positive cells are seen in the second arches (arrows) of twist1a-MO and twist1b-MO embryos. White arrowheads denote the developing ear. (D–G) Confocal projections of fli1a:GFP; sox10:dsRed doubly transgenic embryos at 28 hpf show normal fli1a:GFP expression in un-injected control, twist1a-MO, and twist1b-MO embryos and loss of fli1a:GFP arch expression in twist1a/1b-MO embryos. Arrows indicate fli1a:GFP vascular expression which is unaffected in twist1a/1b-MO embryos. (H–L) Skeletal staining shows malformed mandibular and hyoid skeletons in twist1a-MO and twist1b-MO embryos compared to un-injected controls. In addition, co-injection of a Twist1b mRNA not targeted by the MOs partially rescued the head skeleton of twist1a/1b-MO embryos (n = 24/24), whereas co-injection of a control kikGR mRNA never rescued (n = 0/11). Scale bars = 50 µm. (TIF) [file pgen.1002710.s001.tif]

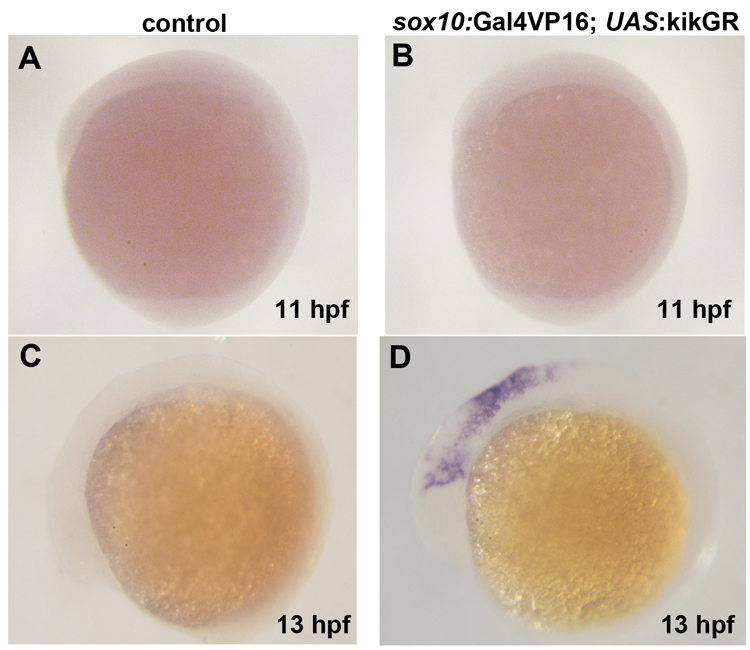

Supplement: Figure S2 — Time-course of sox10:Gal4VP16-dependent transgene expression. (A–D) In situs for kikGR mRNA in sox10:Gal4VP16; UAS:kikGR embryos show transgene expression in migratory CNCCs at 13 hpf but not in pre-migratory CNCCs at 11 hpf. sox10:Gal4VP16 control embryos show no expression. (TIF) [file pgen.1002710.s002.tif]

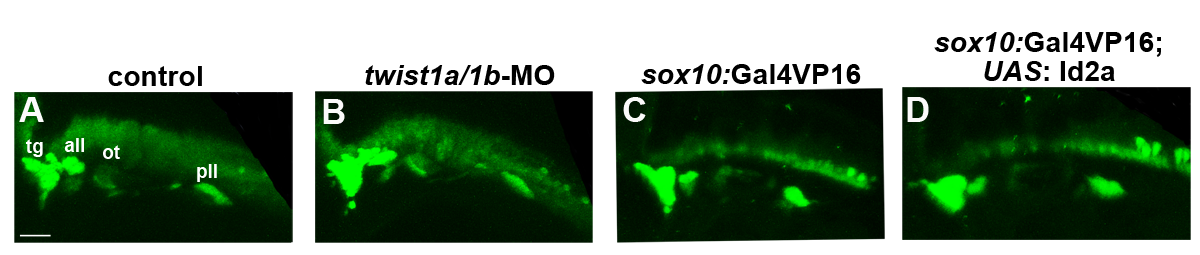

Supplement: Figure S3 — Cranial ganglionic neurons are unaffected in twist1a/1b-MO and Id2a misexpression embryos. (A–D) Confocal projections of anti-HuC/D immunofluorescence show neurons of the trigeminal (tg), anterior lateral line (all), otic (ot), and posterior lateral line (pll) ganglia at 36 hpf. No major differences in the pattern of anti-HuC/D staining was observed between uninjected control (n = 6), twist1a/1b-MO (n = 6), sox10:Gal4VP16 only control (n = 9), and sox10:Gal4VP16: UAS:Id2a (n = 8) embryos. Scale bar = 50 µm. (TIF) [file pgen.1002710.s003.tif]

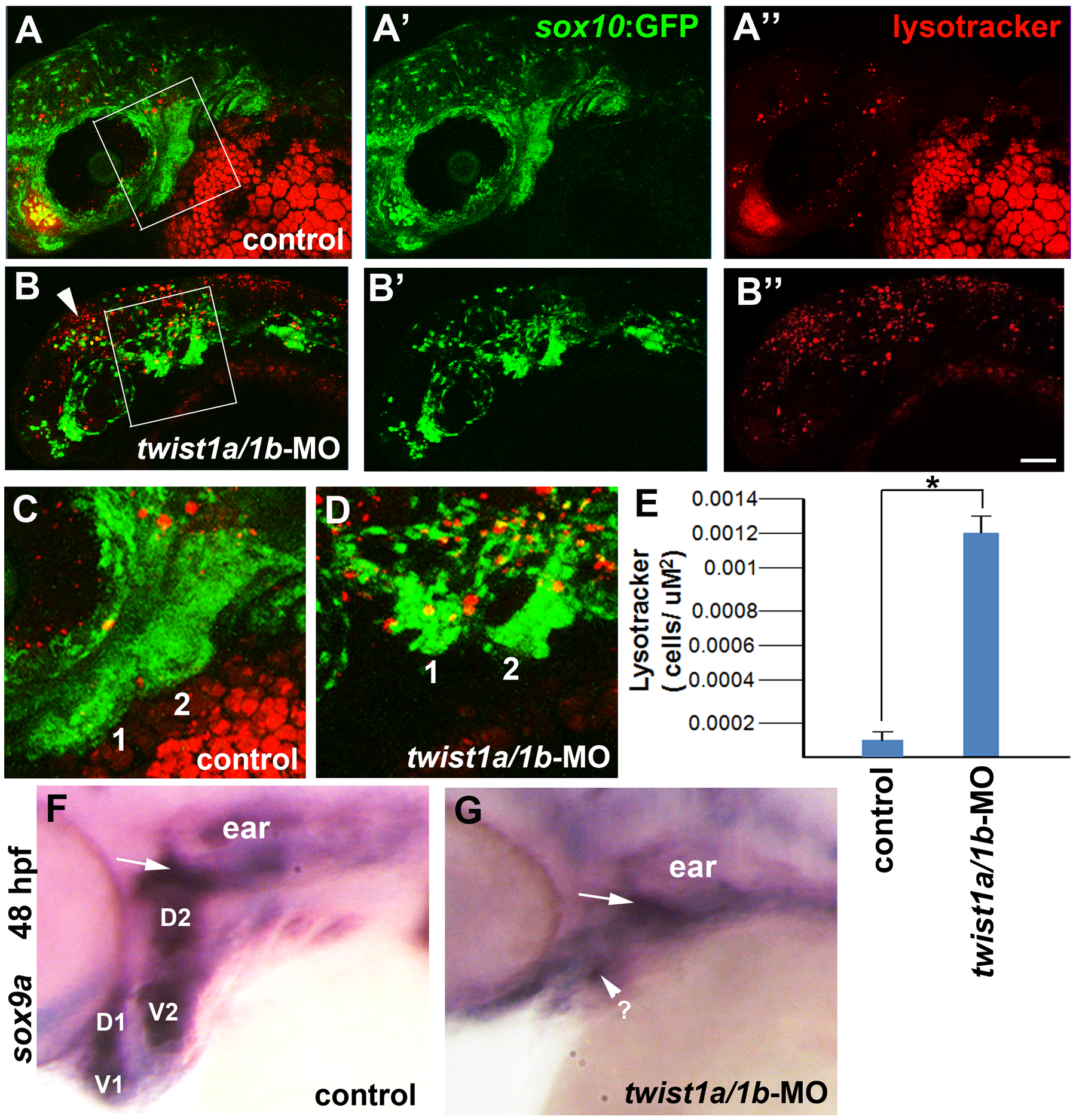

Supplement: Figure S4 — Cell death and sox9a expression in twist1a/1b-MO embryos. (A–D) Confocal projections of Lysotracker Red staining in 36 hpf sox10:GFP transgenic embryos show increased cell death in the pharyngeal arches (shown in high-magnification views in C and D from the boxes in A and B) in twist1a/1b-MO-injected embryos (n = 6) compared to un-injected controls (n = 6). Arrowhead shows increased cell death in more dorsal CNCCs as well. Scale bar = 50 µm. (E) Quantification of Lysotracker-positive cells per arch area. Mandibular and hyoid arches were used for the analysis. Asterisk indicates statistical significance using a Tukey-Kramer HSD test (α = 0.05). (F,G) In situ hybridizations for sox9a at 48 hpf show very reduced expression in the pharyngeal arches of twist1a/1b-MO embryos (n = 5/5) compared to uninjected controls (n = 0/12). Dorsal (D) and ventral (V) pre-chondrogenic domains of the mandibular (1) and hyoid (2) arches are shown for the control. sox9a expression in the pre-chondrogenic domain of the mesoderm-derived otic capsule cartilage (arrows) was less affected in twist1a/1b-MO embryos. (TIF) [file pgen.1002710.s004.tif]

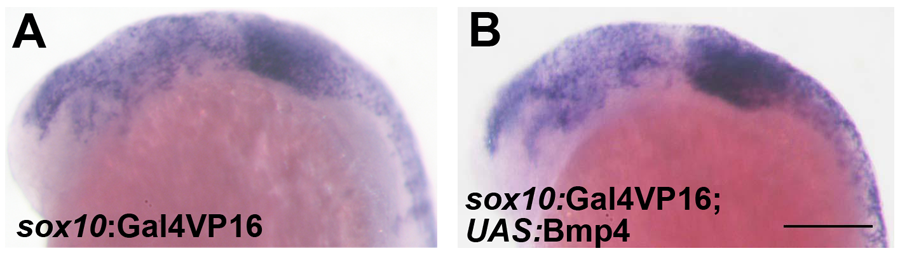

Supplement: Figure S5 — Induction and early migration of CNCCs is unaffected by Bmp4 misexpression. In situs for sox10 at 15 hpf show no difference in early migrating CNCCs between sox10:Gal4VP16; UAS:Bmp4 embryos (n = 5) and sox10:Gal4VP16 only controls (n = 12). Scale bar = 50 µm. (TIF) [file pgen.1002710.s005.tif]

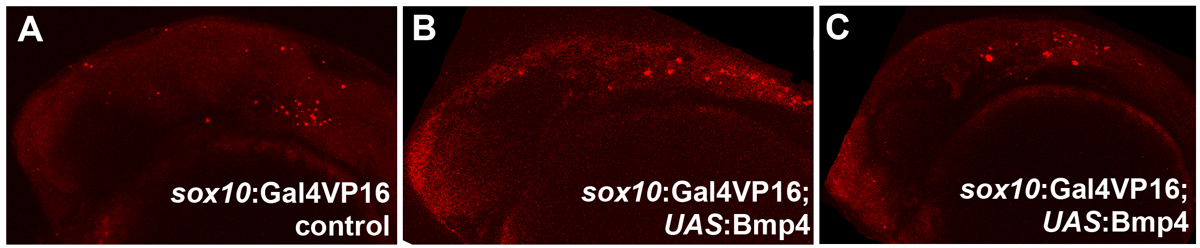

Supplement: Figure S6 — Cell death analysis in sox10:Gal4VP16; UAS:Bmp4 embryos. Compared to sox10:Gal4VP16 only controls (n = 8), Lysotracker staining reveals no major increase in cell death at 24 hpf in sox10:Gal4VP16; UAS:Bmp4 embryos (n = 8). Scale bar = 50 µm. (TIF) [file pgen.1002710.s006.tif]
